# Molecular Mechanism of Protein Arginine Deiminase 2: A Study Involving Multiple Microsecond Long Molecular Dynamics Simulations

**DOI:** 10.1021/acs.biochem.2c00158

**Published:** 2022-06-23

**Authors:** Erdem Cicek, Gerald Monard, Fethiye Aylin Sungur

**Affiliations:** † Informatics Institute, Computational Science and Engineering, Istanbul Technical University, TR-34469 Istanbul, Turkey; ‡ Université de Lorraine, CNRS, LPCT, F-54000 Nancy, France

## Abstract

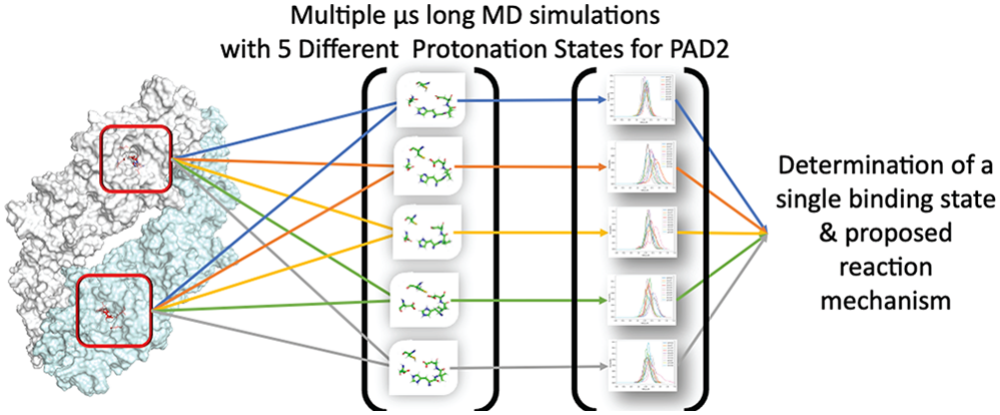

Peptidylarginine deiminase 2 (PAD2) is a Ca^2+^-dependent enzyme that catalyzes the conversion of protein arginine residues to citrulline. This kind of structural modification in histone molecules may affect gene regulation, leading to effects that may trigger several diseases, including breast cancer, which makes PAD2 an attractive target for anticancer drug development. To design new effective inhibitors to control activation of PAD2, improving our understanding of the molecular mechanisms of PAD2 using up-to-date computational techniques is essential. We have designed five different PAD2–substrate complex systems based on varying protonation states of the active site residues. To search the conformational space broadly, multiple independent molecular dynamics simulations of the complexes have been performed. In total, 50 replica simulations have been performed, each of 1 μs, yielding a total simulation time of 50 μs. Our findings identify that the protonation states of Cys647, Asp473, and His471 are critical for the binding and localization of the *N*-α-benzoyl-l-arginine ethyl ester substrate within the active site. A novel mechanism for enzyme activation is proposed according to near attack conformers. This represents an important step in understanding the mechanism of citrullination and developing PAD2-inhibiting drugs for the treatment of breast cancer.

Highly conserved positively charged histone proteins are primary protein components of chromatin fiber complexes serving as the scaffold for DNA in eukaryotic cells. Structural modifications in histone molecules cause loss of interaction with DNA and other nuclear proteins that affect major chromatin functions like transcriptional activation/inactivation, chromosome packaging, and DNA damage/repair.^1,2^

Such structural modifications belong to a set of post-translational modifications, including phosphorylation, methylation, acetylation, ubiquitination, and citrullination.^3^ Thus, control of the regulation of gene expression within such a highly compact environment still represents a challenging question in cell biology.^4,5^ Peptidylarginine arginine deiminase (PAD) enzymes, commonly found in mammalian cells, catalyze the hydrolysis of peptidylarginine to peptidyl citrulline in a reaction called deimination or citrullination.^6−8^ PADs are calcium-dependent enzymes that use a nucleophilic cysteine to hydrolyze guanidinium groups on arginine residues to form citrulline (Figure 1).^9^ This reaction results in the loss of a positive charge, thereby affecting protein function and altering protein–protein and protein–nucleic acid interactions.^10,11^

**Figure 1:**
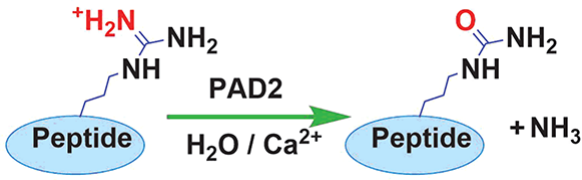
Citrullination reaction catalyzed by PAD2.

The PAD family is composed of five calcium-dependent isozymes (PAD1–4 and PAD6), which share roughly 50% sequence similarity and have different tissue distributions and biological functions.^12,13^ PAD1 has an important role in terminal differentiation of keratinocytes.^14,15^ PAD2 is involved in myelin loss.^16,17^ PAD3 enables hair growth by citrullination of trichohyalin.^18^ PAD4 has been reported to be involved in the regulation of gene expression^19,20^ and in the formation of extracellular DNA traps.^21^ A role in reproduction has been suggested for PAD6.^22^

PAD enzymes have garnered significant attention over the past several years with regard to their dysregulated activity in cancer and involvement in a number of inflammatory (e.g., multiple sclerosis, rheumatoid arthritis, and ulcerative colitis) and autoimmune (lupus) diseases.^7,10,13,23^ Although it is unclear how PADs contribute to such a disparate number of diseases, common links include a role for PAD4 in promoting neutrophil extracellular trap (NET) formation and regulating gene transcription.^24^ Further evidence that upregulated PAD activity plays a role in these various diseases comes from the demonstration that Cl-amidine, a potent pan-PAD inhibitor, or its analogues show efficacy in animal models of cancer,^25,26^ rheumatoid arthritis,^27^ lupus,^28^ thrombosis, spinal cord injury,^29^ and ulcerative colitis.

Although dysregulated PAD4 activity is typically associated with several diseases, more recent work suggests that PAD2 also plays an important role in both extracellular trap formation and gene regulation.^30,31^ Thus, it is possible that PAD4 and PAD2 carry out similar and/or related functions during disease progression. More recently, a detailed ChIP-chip study demonstrated that PAD2 also plays a critical role in ER target gene activation via the citrullination of histone H3Arg26 at ER target gene promoters.^32^ Additionally, it was found that PAD2 expression is highly correlated with HER2 expression across more than 60 breast cancer cell lines.^30^ From a therapeutic standpoint, 75% and 15% of all breast cancers are ER and HER2+, respectively. Given that PAD2 likely plays an important role in the biology of both ER and HER2+ lesions, these observations suggest that PAD2 could represent a therapeutic target for 85–90% of all breast cancers in humans.^30^ The high-resolution structures of PAD1–4 have been reported.^9,33−35^ The catalytic activity for PADs is known to be regulated by calcium ions through conformational changes, including the appropriate positioning of the catalytic cysteine in the active site cleft. Slade et al. have determined the X-ray structures of the apo and holo states for PAD2 (27 in total), including the wild type, and structures with mutations on the calcium binding residues.^34^ The role of the six calcium ions and the activation of PAD2 has been revealed by these successives X-ray structures.

Experimental studies have identified the residues that play a role in the catalytic activity in addition to the mechanistic differences between PAD2 and PAD4.^36^ The catalytic mechanism suggested for PAD2 by Dreyton et al. (see Figure 2) starts with the attack of a nucleophilic Cys647 on the guanidinium carbon of the substrate arginine while His471 protonates the guanidinium group to form the *S*-alkyl tetrahedral intermediate, followed by the departure of an ammonia molecule. In the second part, His471 acts as a general base and activates the water molecule for a nucleophilic attack forming the second intermediate. The reaction is concluded by the formation of the citrullinated product.^36^

**Figure 2:**
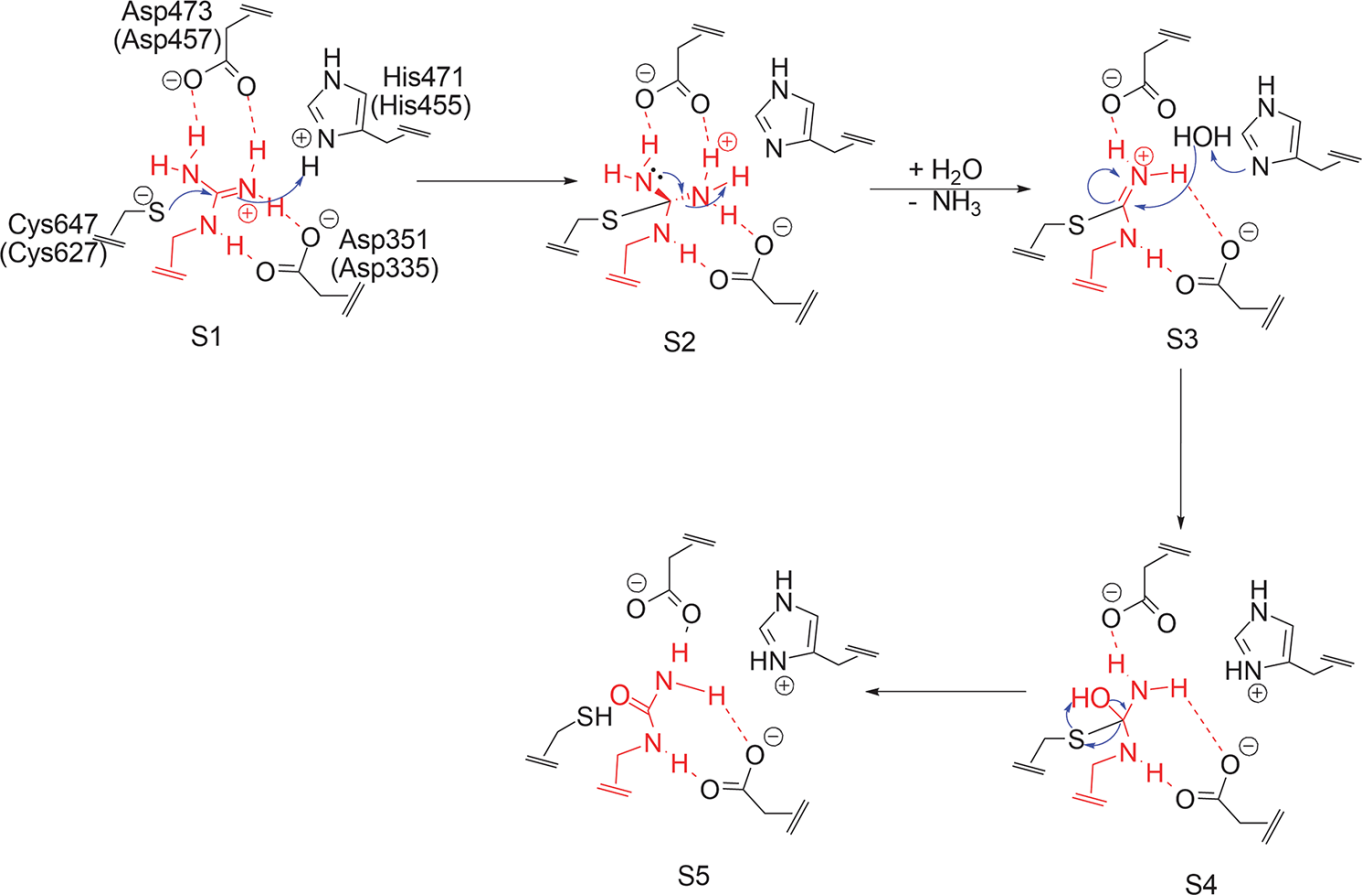
Experimentally proposed catalytic mechanism of PAD2 (from ref (36)).

When proteins are modeled using molecular dynamics (MD), obtaining a correct sampling of their different conformations can be quite a challenge. Given the size of the systems (i.e., the number of degrees of freedom), many configurations must be generated to provide a statistical representative sample. This is usually sought by simulating (very) large MD trajectories. The conjunction of these two requirements, large systems and long trajectories, adds up to yield computationally intensive simulations. In practice, the limited time scale of a protein MD leads to a sampling problem.^37,38^ To overcome it, increasing the simulation time and performing multiple independent simulations are possible solutions.^39^ Recently, simulations of multiple copies with different initial parameters of the same system (e.g., initial speeds, hardware differences, shift multiplication accuracy, etc.) have been preferred over single long simulations to obtain reproducible results.^38,40−43^

In this work, multiple independent MD simulations for the enzyme PAD2 complexed with *N*-α-benzoyl-l-arginine ethyl ester (PAD2–BAEE) were performed to understand the homodimer complex dynamics and particularly its active sites. In this context, five different PAD2–BAEE systems were designed to analyze the effect of protonation state alterations on the active site and ligand binding. For each system, 1 μs long independent MD simulations were performed for 10 independent replicas. The results enabled us to determine the protonation state of the catalytic cysteine and the role of the active site histidine and to elucidate the possible initial attack structures based on the near attack conformation approach.

## Computational Details

The crystal structure of protein arginine deiminase 2 (PAD2) in its holo form, with a resolution of 3.02 Å, was obtained from the Protein Data Bank (PDB entry 4N2C).^34^ The biological assembly of this protein consists of two identical monomers of 690 amino acid residues each. Six Ca^2+^ ions per monomer are included in the system to facilitate enzyme activation.^34^ The monomers are labeled hereafter as chain A and chain B. This nomenclature will be used to distinguish between the monomers during the MD analysis. *N*-α-Benzoyl-l-arginine ethyl ester (BAEE) with known activity and affinity values from experimental studies by Dreyton et al.^36^ was selected as the ligand due to its high selectivity for PAD2.

### Generating the Enzyme–Substrate Complex

**Figure 3:**
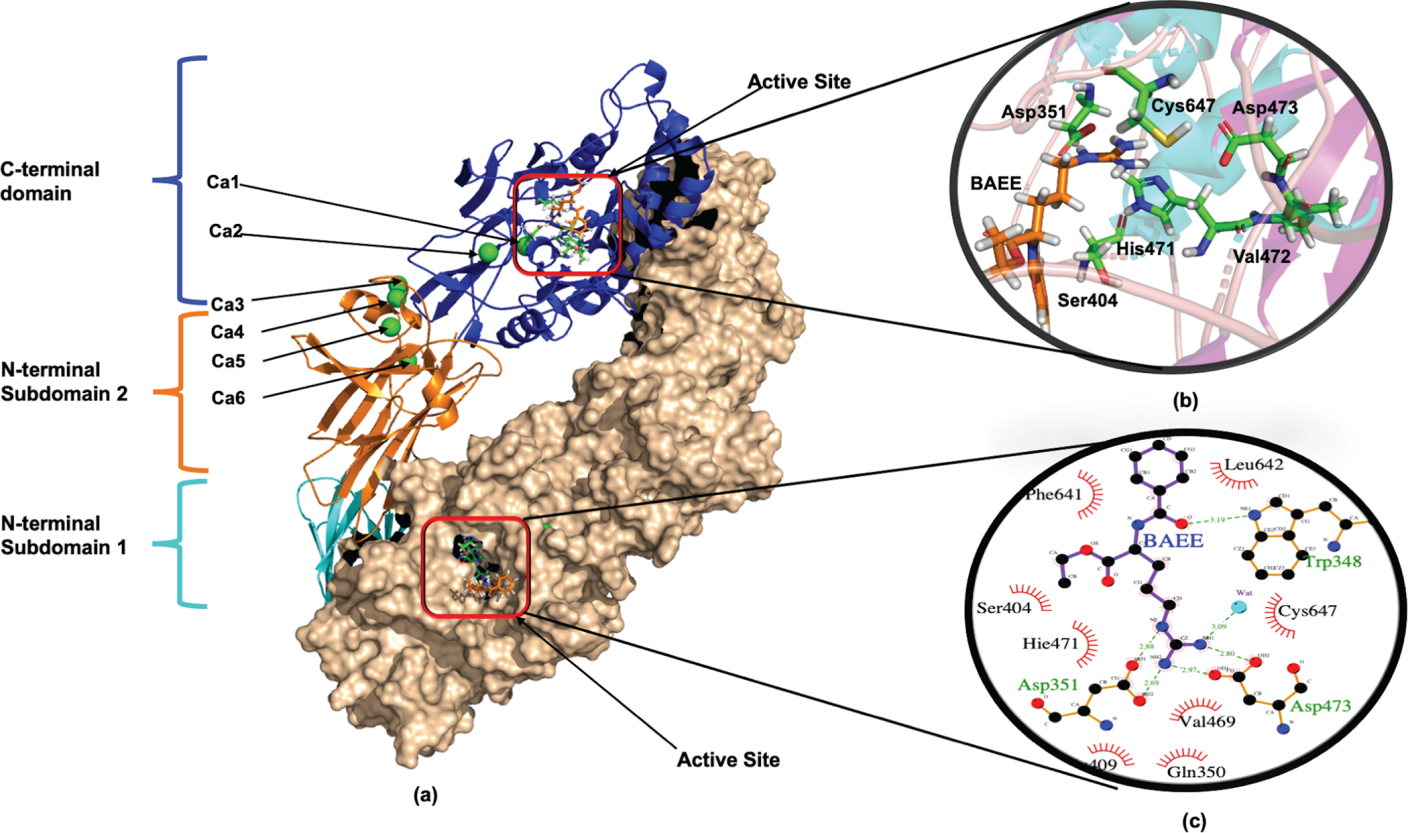
Structure of the PAD2–BAEE complex. (a) Chain A (cartoon) and chain B (molecular surface). (b) Close-up of the initial docking pose. (c) Two-dimensional LigPlot+ representation of the interactions in the active site of BAEE-bound PAD2.

The molecular docking program AutoDock 4.2^44^ was employed to generate docked conformations of BAEE as the ligand and 4N2C as the macromolecule host. The following docking protocol was applied. (i) The box was centered on the sulfur atom of the catalytic cysteine. (ii) The number of grid points was defined at 40 in all three dimensions with the default spacing (0.375 Å). (iii) The receptor was kept rigid while the ligand was allowed to move. (iv) Ten independent runs were performed with a population of 150 and a maximum number of energy evaluations of 2 500 000, with all remaining parameters being kept at their default values. The pose with the lowest binding energy was chosen as the initial ligand configuration and used as an initial structure for the subsequent MD simulation (see Figure 3). It contains many interactions already found for similar structures published for PAD4 (PDB entries 5N0M, 5N0Y, 5N0Z, and 5N1B(45)). The substrate arginine is inserted into the active site cleft, between His471 and Cys647; Asp473 interacts by hydrogen bonds with the guanidinium fragment as well as Asp351 (see Figure 3c).

### Protonation States of Active Side Residues

The initial crystallographic structure from which we have built our initial system has been determined at a resolution of 3.02 Å. With such a resolution, the hydrogen atoms are of course missing in the reported PDB structure. Adding the right number of hydrogen atoms, especially around and inside the active site, represents a crucial step of this molecular modeling. To identify which protonation state(s) should be envisaged for PAD2 molecular dynamics, we have compared experimental p*K*_a_ values and suggested mechanisms reported in the literature with Propka3^46^ computations.

First, Dreyton et al. have evaluated the p*K*_a_ of Cys647 at ∼8.2 by measuring the rates of inactivation of PAD2 as a function of pH.^36^ From their experimental results, they have suggested a catalytic mechanism that starts with a thiolate that proceeds to a nucleophilic attack on the guanidinium carbon of the substrate arginine. Within this step, a positively charged His471 acts as a general acid, while Asp473 and Asp351 provide electrostatic stabilization.

We report in Table 1 estimations of p*K*_a_ values by Propka3 for three different systems: the apoenzyme (PDB entry 4N2C), the holoenzyme (PDB entry 4N2C), and the ligand-bound enzyme (our docking structure). The computationally estimated p*K*_a_ value for Cys647 is much higher than the value reported by Dreyton et al.^36^ From Propka3, this would be due to a strong desolvation shift of the “buried” Cys647 that is not compensated by the charge–charge interaction with the guanidinium fragment upon binding. This indicates that we should envisage for the initial stage of the catalysis mechanism that Cys647 could be in a neutral form instead of a thiolate form. Adding a ligand inside the active site pocket yields a sharp decrease in the estimated p*K*_a_ of His471. This would indicate that the histidine would remain neutral and therefore could not act as a general catalyst. To clarify this discrepancy between the experimental and computational results, we think that multiple protonation states for His471 should be included in our MD models. While Propka3 predicts an acid behavior for Asp351, reinforced by our docking study that suggests a stabilizing role through hydrogen bond interactions with the substrate, Asp473 presents a high p*K*_a_ value (>7) in the case of the holoenzyme. In the docking structure, a possible hydrogen bond pattern between Cys647 and Asp473 is present. This suggests that this residue could be involved in proton transfer.

**Table 1:** p*K*_a_ Values of the Apoprotein, Holoprotein, and Complex Forms Estimated by Propka3

	4N20 (apo)	4N2C (holo)	4N2C–BAEE (complex)
Cys647	14.25	16.01	15.38
Asp473	4.26	7.76	4.86
Asp351	3.23	4.10	6.19
His471	6.96	4.70	0.46

**Figure 4:**
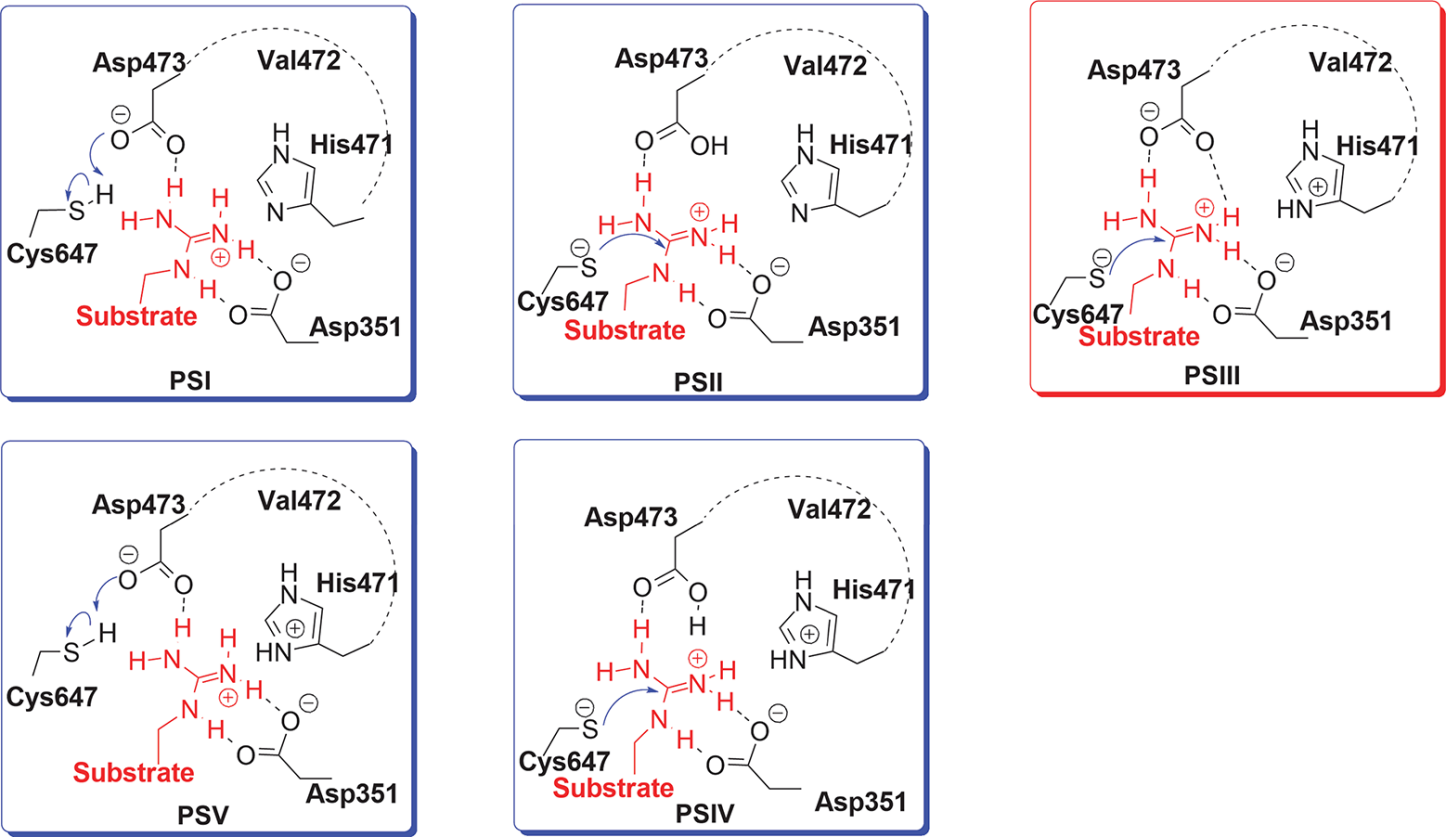
Constructed models based on the different protonation states of the active site residues. Note that PS-III corresponds to the initial stage of the catalytic mechanism proposed by Dreyton et al.^36^

In light of these findings, we have decided to create MD models that examine the different protonation states of the four main residues in the active site. The five different models that we have simulated are presented in Table 2. They are labeled hereafter PS-I–PS-V. Protonation states for Cys647, His471, and Asp473 vary, while Asp351 is always considered to be in a deprotonated form. Figure 4 depicts the difference in active site protonation states for the different models of this study. PS-I represents an initial stage where Cys647 could be activated by Asp473. In PS-II, the initial stage of the catalytic reaction is an already deprotonated Cys647 with a neutral Asp473. PS-III corresponds to a papain-like initial stage, as suggested by Dreyton et al.,^36^ where Cys647 is deprotonated and His471 is positively charged. PS-IV is similar to PS-II, but His471 is now doubly protonated. Finally, in PS-V, both Cys647 and His471 are protonated; therefore, only Asp473 can act as a proton acceptor. Except for these four residues, the protonation states of all remaining residues are identical for all MD models.

**Table 2:** Charges of the Active Site Residues for Each Model System

system	Cys647	Asp473	His471	Asp351
PS-I	0	–1	0	–1
PS-II	–1	0	0	–1
PS-III	–1	–1	+1	–1
PS-IV	–1	0	+1	–1
PS-V	0	–1	+1	–1

### Molecular Dynamic Simulations

Each system was built with the tleap module of AMBER18.^47^ From the initial complex structure generated with AutoDock, the systems were protonated according to their respective definitions (e.g., PS-I, PS-II, etc.). Proteins and water molecules were described using the ff14SB29^48^ and TIP3P^49^ force fields, respectively. Periodic boundary conditions were applied by filling an isometric truncated octahedral box containing one complex system (a PAD2 dimer and two ligands, one for each active site) with water molecules. The minimum distance between any protein atom and the edge of the periodic box was set to 18 Å. The neutrality of the systems was imposed by adding the right number of chloride ions depending on the given system. For each system, the total number of solute atoms was around 20 500 atoms, while the number of water molecules was approximately 86 500. Energy minimization and MD simulation runs were performed using the GPU-supported pmemd module in AMBER18.^47^ The particle mesh Ewald summation technique was used with the default 8 Å cutoff, and the SHAKE algorithm^50^ for bonds involving hydrogen atoms was applied in addition to hydrogen mass repartitioning (HMR).^51^

The samples were equilibrated in three consecutive steps. First, 100 ps of molecular dynamics was performed in the *NPT* ensemble at a temperature of 10 K with harmonic positional restraints on the heavy atoms of the protein–ligand parts (50 kcal mol^–1^ Å^–2^). The Andersen thermostat was used, and velocities were rescaled at 10 K every 10 steps to ensure a rapid decrease in the potential energy to an approximate local minimum. Then the systems were linearly heated to 300 K in the *NPT* ensemble during 2100 ps of molecular dynamics while the harmonic positional restraints on the heavy atoms of the protein–ligand parts were maintained. The increase in temperature was ensured again by using the Andersen thermostat with a velocity rescaling every 10 steps to the appropriate temperature. For this first two steps, constant-pressure dynamics at 1 bar was applied with isotropic position scaling and controlled by a Monte Carlo barostat with volume change attempts every 100 steps. The time step was 0.002 fs. Finally, the harmonic potential restraints were linearly lifted during a 50 ns molecular dynamics, the value of the restraints being decreased by 1 kcal mol^–1^ Å^–2^ every nanosecond. This molecular dynamics and the production molecular dynamics were performed using the *NVT* ensemble, and the temperature was controlled by a Langevin thermostat with a collision frequency γ of 1.0 ps^–1^. The time step was 0.004 fs. Production runs of 1 μs were carried without restraints in the *NVT* ensemble, and snapshots were saved every 40 ps (hence 25 000 snapshots per MD simulation).

For each of the five different constructed MD systems within the scope of this study (PS-I–PS-V), 10 independent simulations were performed using different initial random seeds. The 50 replica simulations, each of 1 μs, represent a total simulation time of 50 μs as well as 100 different active site trajectories to analyze.

### Analysis Methods

All analyses were performed using the cpptraj module of AMBER18.^47^ In addition to various distance, angle, and dihedral analyses, root-mean-square deviations (RMSDs) were computed to assess the stability of the simulations. The interactions between Cys647 and water molecules were computed using radial distribution functions (RDFs). The solvent accessible surfaces of the protein itself (*P*), the ligand itself (*S*), and the protein–ligand complex (PS) were evaluated using the surf command by incrementing the van der Waals radii by 1.4 Å. The surface of contact (SC) between the protein and the ligand was simply defined by
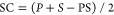
1

All scripts and extended data analysis can be found in the Supporting Information.

## Results and Discussion

Ten multiple 1 μs MD simulations using random initial velocities were applied to each system to enhance conformational samplings of the protein–ligand complexes. The presence of different types of interactions and movements of the protein domains obtained from the 10 replicas for each system enabled us to engage in a deeper discussion related to the structure and dynamics of these protein–ligand complexes.

### Stability of the Simulated Systems

**Figure 5:**
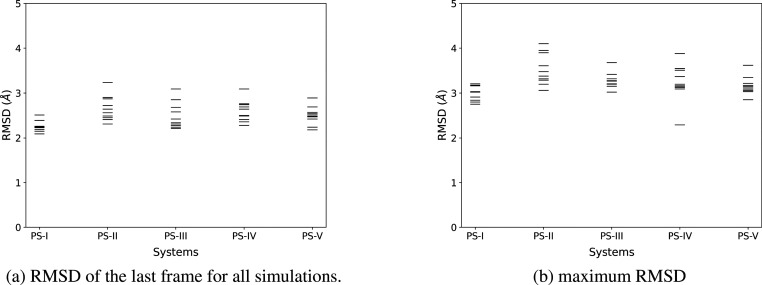
(a) RMSDs of the last frame and (b) maximum RMSDs for all 10 simulations and all five systems.

A first picture of the stability of the systems can be provided by the RMSDs of the atomic positions of the backbone atoms (C_α_) for the dimer during the course of the 1 μs simulations with respect to the reference crystal structure. All RMSD variations for all simulations are given in the Supporting Information. As a summary, we represent in Figure 5 the maximum RMSD as well as the RMSD of each last frame of the 10 independent MD runs for the five considered systems. All observed RMSDs range between 2 and 3 Å. This indicates the good stability of the systems during the MD runs.

### Is the Active Site Cysteine Preactivated?

Two different mechanisms were proposed for the conversion of a peptidyl-arginine into peptidyl-citrulline by different isoforms of PADs. The experimental and theoretical studies have revealed that the active site of PAD4 involves a Cys645 (Cys647 in PAD2) in thiolate form in the apo and holo structures stabilized by a protonated His471 just like the thiolate–imidazolium pair in papain.^52^ The reaction mechanism is denoted as reverse protonation.^53^ In contrast, a substrate-assisted mechanism has been proposed in the case of PAD2. Experimental studies suggest that the p*K*_a_ of the active site cysteine residue (Cys647) is shifted after the binding of the 2-chloroacetamidine molecule that is analogous to the positively charged substrate guanidinium group.^36^ Meanwhile, the active site histidine (His471) is suggested to be protonated. Variations on the protonation states of an enzyme active site have consequences on its possible reaction mechanisms; this is why it is important, before any further mechanistical studies, to clearly assess the protonation states of the active site residues.^54−57^

**Figure 6:**
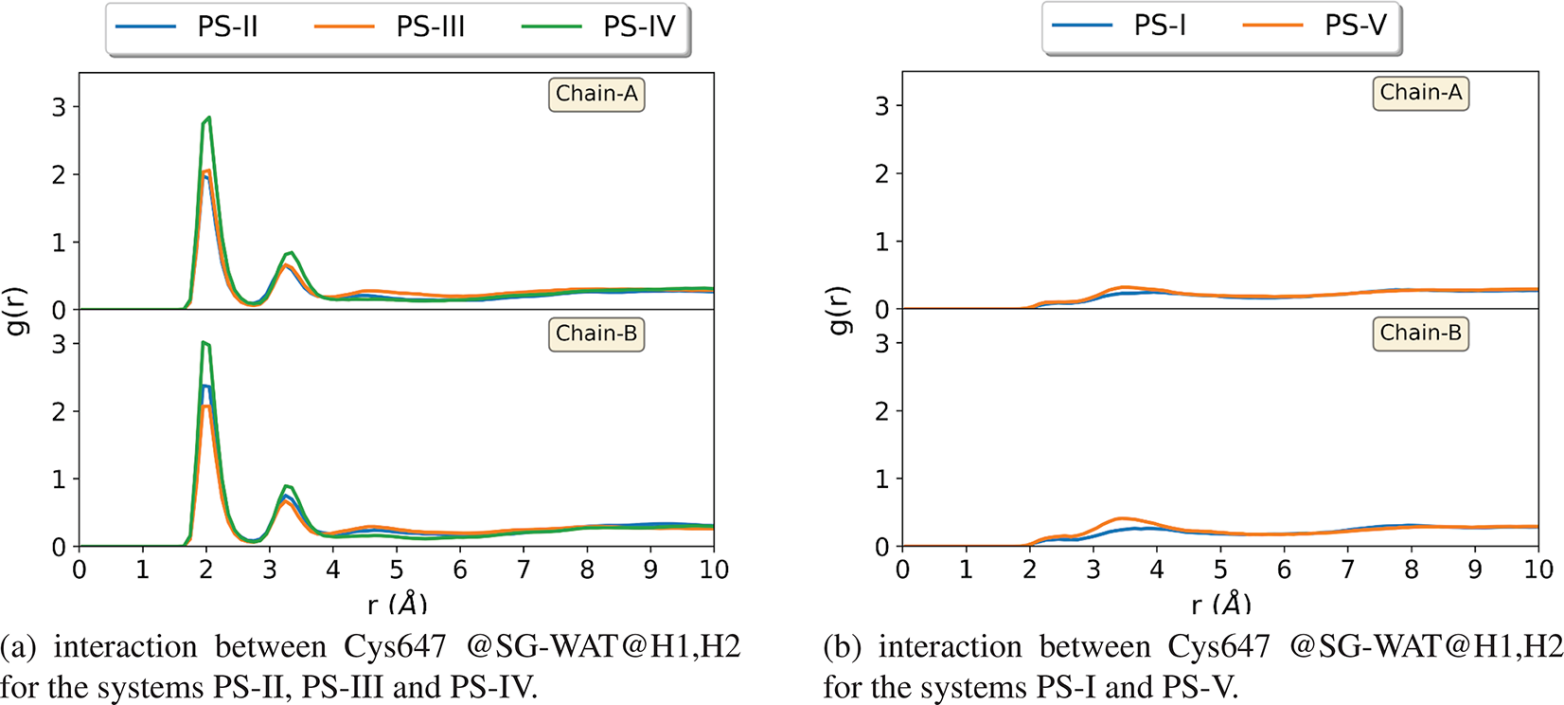
Radial distribution function, *g*(*r*), between the sulfur atom of Cys647 (Cys647@SG) and water hydrogen atoms for all five considered systems.

To elucidate the structure of the Michaelis complex, five different systems were designed on the basis varying protonation states. As presented in Table 2, for the PS-II–PS-IV systems, the catalytic cysteine (Cys647) is deprotonated, whereas in PS-I and PS-V, it must be activated through a proton transfer to Asp473. As a first analysis, we have investigated the interactions between the Cys647 side chain and solvent water molecules for all PAD2–BAEE samples by RDF analysis. According to the radial distribution function [*g*(*r*)] patterns depicted in Figure 6, the probability of finding a water molecule at a certain distance *r* from the S_γ_ atom varies depending on the protonation state of Cys647. A strong peak around 2 Å indicates a hydrogen bond interaction between the sulfur atom and water. Moreover, the average number of water molecules around this sulfur atom during the simulations can be estimated by the integration of RDF curves until the first minima. The *g*(*r*) functions in systems having a deprotonated cysteine (systems PS-II–PS-IV) are depicted in Figure 6a. The estimated numbers of water molecules obtained by the integrations of the *g*(*r*) curves are found to be approximately two molecules for PS-II and 1.5 molecules for PS-III and PS-IV. This shows that the negatively charged Cys647 side chains are surrounded by water molecules that prevent the attack of the sulfur atom on the carbon atom of the substrate guanidinium. On the contrary, the RDF patterns given in Figure 6b for the PS-I and PS-V systems show no typical hydrogen bond peak. This indicates that when the Cys647 side chain is neutral, the number of water molecules is much smaller in the active site and they do not form hydrogen bond interactions with Cys647. Integrations of *g*(*r*) until 2.75 Å for both systems indicate that only ∼0.3 water molecule is in the vicinity of the sulfur atom.

These results can be interpreted in two ways. First, the presence of strong water interactions between the deprotonated Cys647 and approximately two water molecules in average for systems PS-II–PS-IV prevents Cys647 from attacking the substrate. These systems can therefore be considered as nonreactive. Second, the usage of a molecular mechanics force field here does not allow proton transfer. It seems that the strong hydrogen bond interaction could be seen as the mark of a potential proton transfer between Cys647 and solvent water, which would indicate that the p*K*_a_ of Cys647 is much larger than 7 (i.e., the stable form of Cys647 tends to a thiol form rather than a thiolate one).

### Role of Histidine

Radial distribution function analysis revealed that a neutral Cys647 should be favored in the PAD2 system for the Michaelis complex structure. Hence, we will rule out systems PS-II–PS-IV in the rest of this analysis and focus now on only PS-I and PS-V. The difference between the two latter systems comes from His471. In PS-I, His471 is neutral, while it is protonated in PS-V.

To gain further insight into the differences between the two systems, substrate–active site surface contact calculations, trajectory analysis, and a linear interaction energy (LIE) analysis between the His471 residue and the substrate molecule have been performed.

Visual inspections of the MD reveal some major differences between the two types of systems in the behavior of the substrate. To illustrate these differences, we have computed the contact surface area using eq 1 with *P* being the solvent accessible surface (SAS) around the set of the four active site residues (taken alone), *S* the SAS of the BAEE substrate, and PS the SAS of the complex.

**Figure 7:**
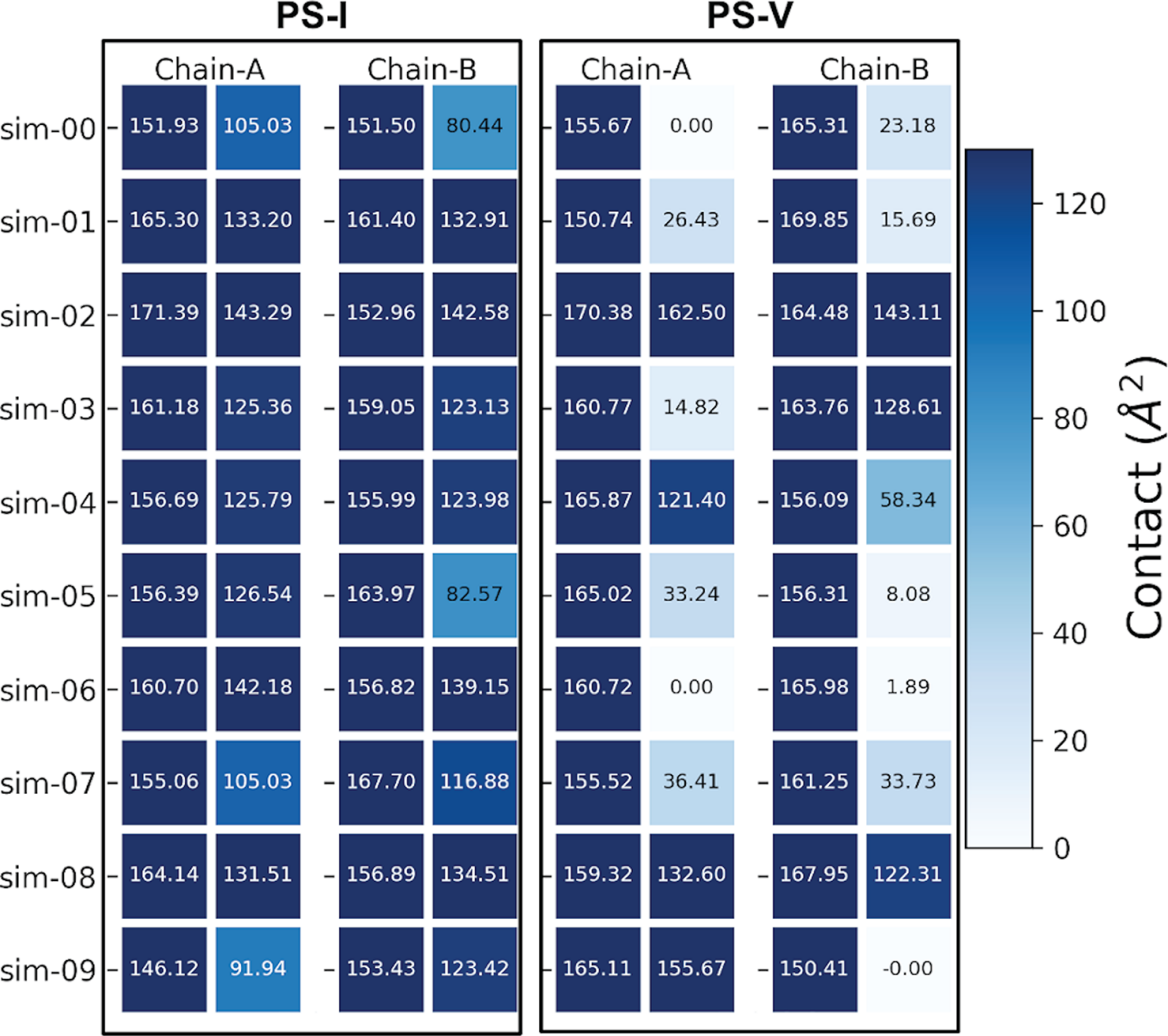
Surface contact area, in square angstroms, between the BAEE substrate and the active site residues for both monomer chains (A and B) of the PS-I (left) and PS-V (right) systems. For each chain, the left column represents the maximum surface contact area and the right column represents the surface contact area of the last MD frame.

The calculated values for the surface contact areas for the two systems, 10 simulations, and two monomers are illustrated in Figure 7. The higher the number (and the more blue), the larger the contact area between the substrate and the active site. For each chain, the first column represents the average contact while the second column represents the surface contact at the end of the microsecond simulations. The stricking point here is that, for PS-V, the surface contact area is mainly close to zero at the end of the simulations. This means that the substrate has left the active site and bears no contact with it anymore. Roughly, a surface contact area of <60 Å^2^ can be considered as a substrate that does not maintain contact with the main atoms of the catalytic site, while a value of >100 Å^2^ can be interpreted as a good attachment of the substrate inside the active site. In PS-I, large surface contact areas are maintained for all simulations and for both chains. In contrast, with a protonated His471 in PS-V, the contact is not maintained for a majority of the complexes. We further extrapolate that for the few PS-V simulations where the substrate stays inside the active site pocket, there would be a great chance that the substrate would depart if the MD were to be extended beyond the current microsecond length. It is noteworthy that, as opposed to the case for PS-V, the substrate remains stuck to the active site for PS-II–PS-IV (see Figure S7), but the solvation of the negative Cys647 diminishes the surface contact area between the host and the ligand.

**Figure 8:**
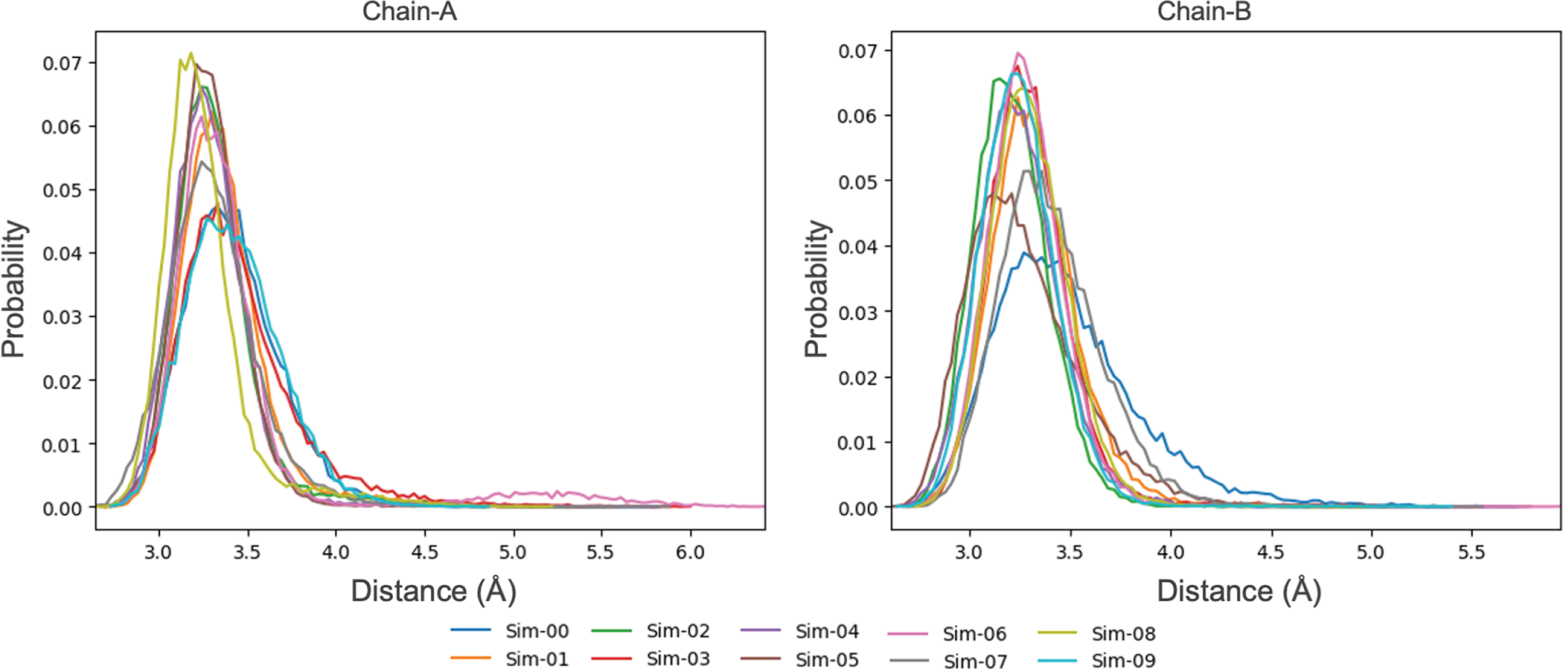
Minimum distance probabilities between His471 and the substrate amino groups for PS-I system subunits A and B.

**Figure 9:**
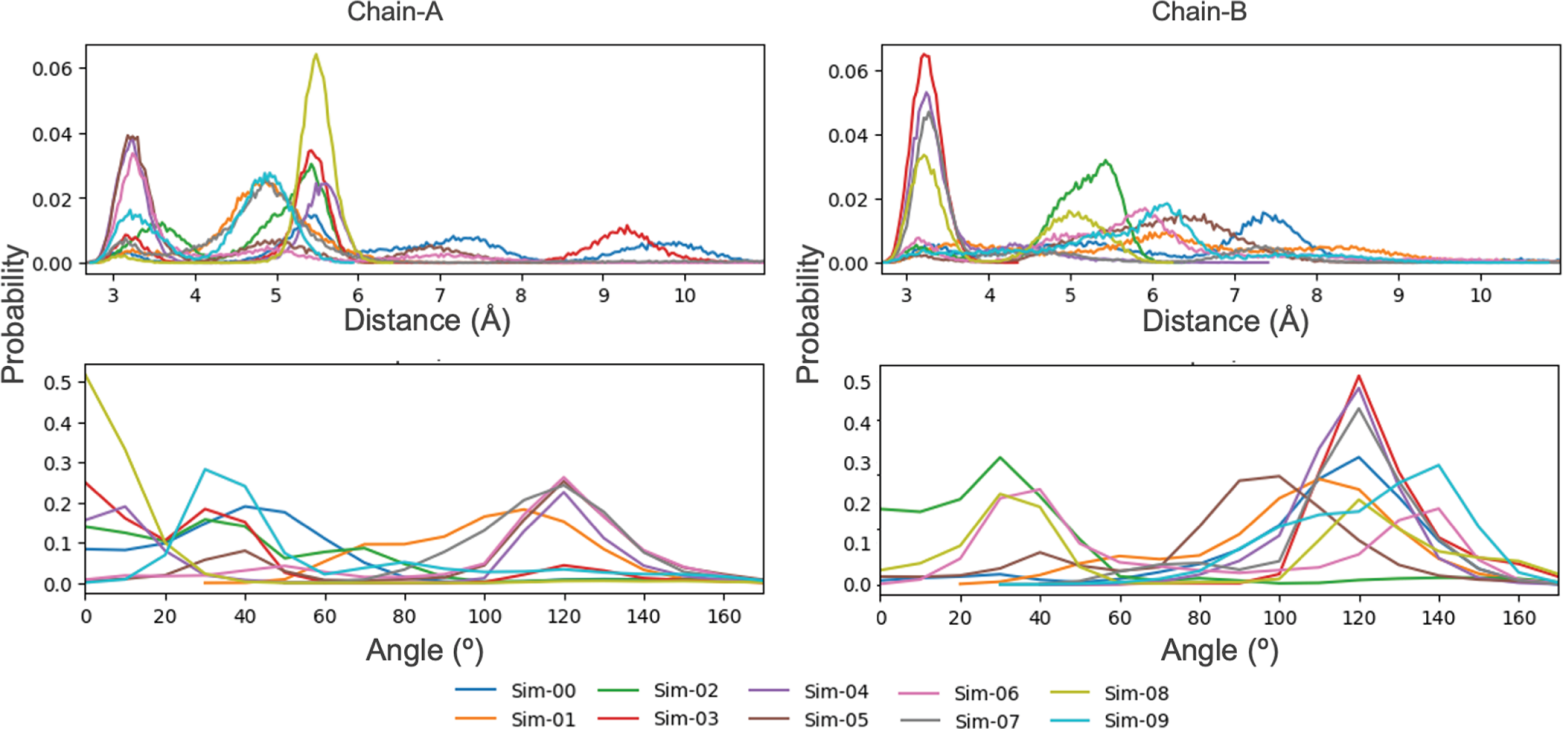
Minimum distance probabilities between His471 and the substrate amino groups (top) and angle probabilities formed by His471@ND1-His471@HD1-Arg623@NH (bottom) for the PS-V system subunits A (left) and B (right).

Another way to illustrate the differences between PS-I and PS-V is to check the distance between the substrate and His471. The minimum distance probabilities between the arginine side chain of the substrate, BAEE, and the imidazole ring of His471 are reported for PS-I and PS-V in Figures 8 and 9, respectively. In the case of a deprotonated His471, the interaction is stable with a peak at ∼3.3–3.4 Å for both monomers. When His471 is protonated, large distances between the imidazole ring and the substrate arginine can be observed, and this confirms a departure of the substrate from the active site during most of the MD trajectories.

Also, the positioning of His471 in PS-V does not favor a proton transfer to the substrate as illustrated in Figure 9 by the computation of the angle between the ND1 and HD1 atoms of His471 and the closest NH atom of the substrate arginine. An angle of ∼180° would favor a proper proton transfer from the imidazole ring of His471 to the guanidinium group of BAEE. For the few MD runs in which the substrate remains inside the active site, this angle has a maximum probability of ∼120°, away from being at the appropriate value to facilitate the proton transfer.

To understand the differences between PS-I and PS-V, and especially to provide a reason for the departure of the substrate when His471 is protonated, we have computed the LIEs between the His471 side chain and the substrate arginine side chain for all 10 independent replicas of the PS-I and PS-V systems. The calculated LIE values are listed in Table 3. A higher affinity between His471 and the substrate in all copies of the PS-I system facilitates the positioning of the substrate in the active site. In contrast, the presence of a repulsive interaction between His471 and the substrate molecule can be seen as the primary reason for the escape of the substrate from the active site of the PS-V system.

**Table 3:** Average Linear Interaction Energies (LIEs) in Kilocalories per Mole between His471 and the BAEE Substrate for Chains A and B of PS-I and PS-V

	PS-I		PS-V	
	chain A	chain B	chain A	chain B
Sim-00	–6.8 (2.1)	–6.3 (2.4)	21.9 (17.5)	22.4 (13.5)
Sim-01	–9.4 (2.6)	–9.5 (1.9)	28.0 (9.0)	20.7 (15.0)
Sim-02	–9.5 (2.4)	–10.3 (1.3)	44.9 (4.3)	41.1 (5.3)
Sim-03	–8.2 (3.2)	–9.7 (2.1)	34.5 (16.5)	49.3 (3.6)
Sim-04	–9.9 (2.1)	–9.9 (1.9)	46.8 (4.2)	45.1 (9.3)
Sim-05	–10.0 (1.6)	–8.0 (3.1)	43.5 (11.0)	20.9 (12.1)
Sim-06	–8.7 (4.3)	–10.0 (1.5)	31.0 (20.5)	26.3 (13.9)
Sim-07	–9.0 (2.8)	–9.7 (1.7)	26.3 (10.3)	42.2 (13.9)
Sim-08	–9.1 (3.3)	–10.0 (1.4)	43.3 (3.3)	47.0 (4.5)
Sim-09	–6.3 (2.2)	–10.3 (1.5)	44.6 (5.7)	21.9 (10.3)

The obtained results suggest that the protonation state of His471 plays a critical role in the proper positioning of the substrate molecule in the active site. Due to the charge distribution inside the active site that creates an electrostatic repulsion between the substrate and His471 within the PS-V system, the substrate molecule is not stable in the active site during most of the simulations. Thus, the PS-V system has also been considered to represent an inappropriate protonation state for PAD2 reactivity and has been eliminated from further analysis.

### Near Attack Conformers

The remaining system, PS-I, is the only system that stabilizes clearly the substrate inside the active site pocket. In this system, BAEE is sandwiched between the neutral catalytic cysteine, Cys647, and the histidine residue, His471, in a neutral form also. This histidine residue is too far from Cys647 and is blocked by BAEE to serve as a general base to activate the nucleophilicity of Cys647. RDF analysis (Figure S17) between the carboxylate oxygen atoms of Asp473 and the hydrogen atoms of the guanidinium fragment of BAEE indicates that, on average, Asp473 makes 0.85 hydrogen bond with BAEE (integration of the RDF until the first minimum at 2.3 Å). Therefore, Asp473 is properly positioned and partially free to abstract, directly or indirectly, the proton from Cys647. Detailed distance analysis shows that the amino groups of the substrate molecule form a stable salt bridge with the embedded Asp351 carboxylate ion during the MD simulations. With this interaction, the guanidinium substrate fragment is properly positioned for Cys647 to attack (Supporting Information).

**Figure 10:**
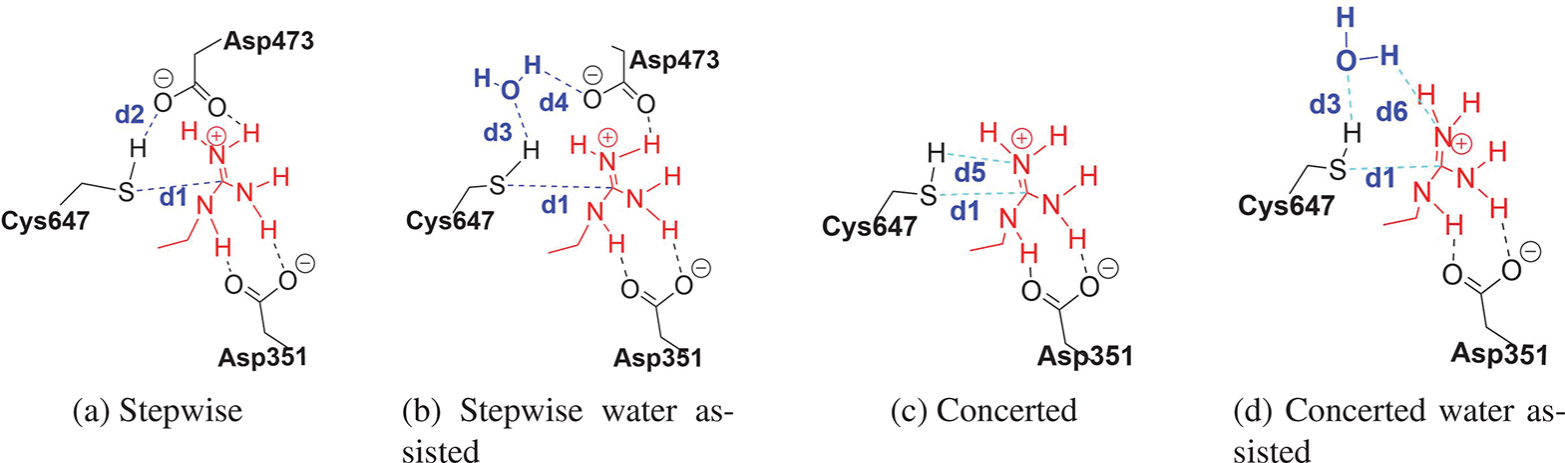
NAC reaction coordinate definitions.

Possible initial reaction mechanisms from PS-I are depicted in Figure 10. The first possible mechanism starts with a direct proton transfer from Cys647 to Asp473 (Figure 10a). The second possible mechanism involves a water-assisted proton transfer to activate Cys647 by Asp473 (Figure 10b). A direct concerted mechanism can be imagined between Cys647 and BAEE. This is a third possible mechanism (Figure 10c). Finally, the attack of Cys647 on BAEE without the assistance of Asp473 could be water-assisted (Figure 10d). To check which mechanisms could be the most plausible, we can use the concept of near attack conformers (NACs) that states that ground state structures tend to adopt favorable conformations that can convert easily to transition states.^58,59^

To analyze the probabilities of having NAC structures in our simulations, we have defined six critical distances based on the four possible initial attacks for the reaction pathway (Figure 10). The active site cysteine, Cys647, should perform a nucleophilic attack to the substrate guanidinium. The distance between Cys647@SG and guanidinium@CZ for the nucleophilic attack is defined as *d*_1_. According to our ongoing QM studies (data not shown), *d*_1_ should be <3.5 Å to reflect a van der Waals contact between the sulfur atom and the guanidinium π orbitals. Distance *d*_2_ reflects the direct proton abstraction of Cys647 by Asp473. Distances *d*_3_ and *d*_4_ defined a water bridge between Cys647 and Asp473 that could facilitate a water-assisted mechanism. These three distances are critical for proton transfer, and a threshold of 2.5 Å was chosen to mimic hydrogen bond character. For the concerted mechanism, an additional distance, *d*_5_, was defined between the sulfur-bound hydrogen and the closest of the guanidinium nitrogen atoms. In conjunction with a proper *d*_1_ distance, a *d*_5_ distance of <2.5 Å is a synonym of NAC for the concerted mechanism. Finally, in the case of a water-assisted concerted mechanism, distance *d*_6_ is defined between the closest hydrogen of the assisting water molecule and the guanidinium nitrogen atoms with a threshold of 2.5 Å.

**Figure 11:**
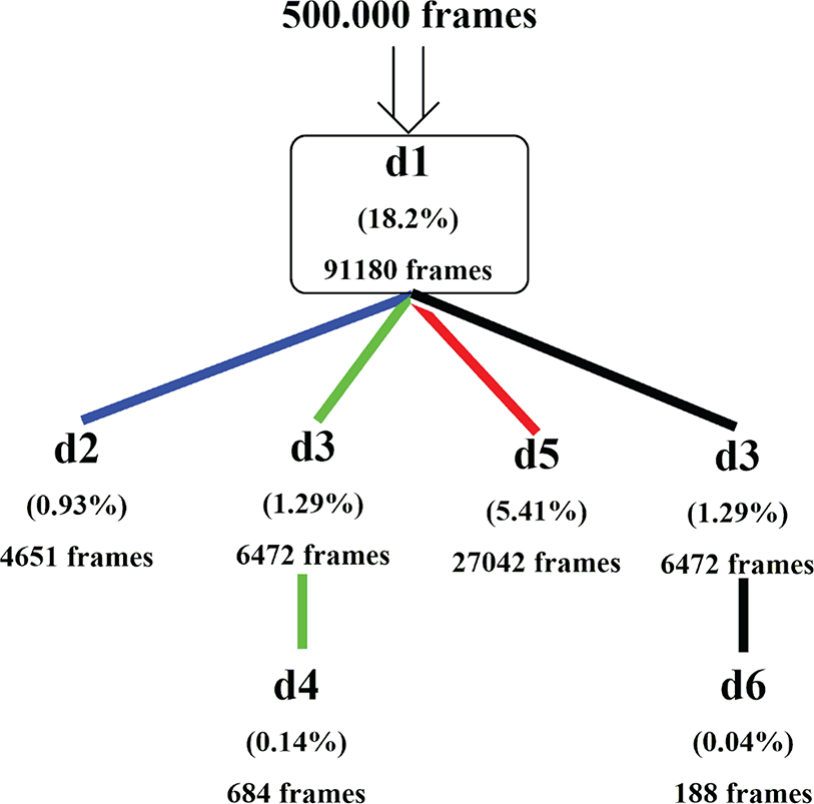
Percentage and number of selected NAC structures based on the predefined distance criteria.

The distance analysis was carried out on 500 000 frames starting with the distance criterion *d*_1_, which is common to all mechanisms. The results are given as the number and percentage of NAC structures in Figure 11; 18.2% of the frames in PS-I contain a short distance between the Cys647 sulfur atom at SG and the center carbon of the guanidinium fragment at CZ. These frames are candidates for a possible initiation of a reaction between Cys647 and BAEE. From these 91 180 frames, 27 042 (5.41%) are compatible with a concerted mechanism. Then, 4651 frames can be regarded as the start of a stepwise mechanism. For the water-assisted mechanism, only 684 and 188 frames are found for the stepwise and concerted mechanisms, respectively.

**Figure 12:**
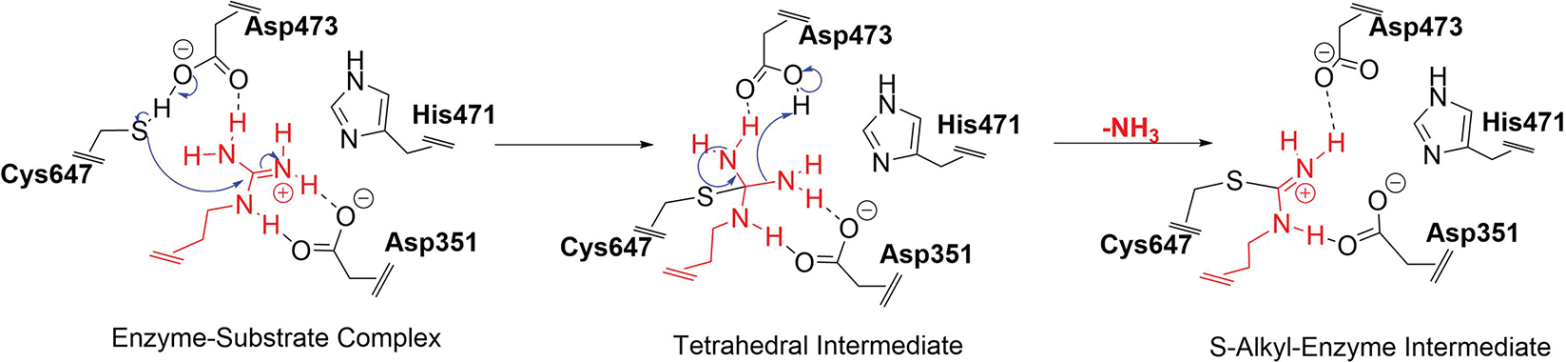
Proposed reaction mechanism for the formation of the S-alkylation intermediate in PAD2.

While NACs have been found for all mechanisms, those who do not involve water assistance are the most prominent. The concerted mechanism that consists of the attack of the sulfur atom on the guanidinium carbon while the sulfur hydrogen is transferred to the nitrogen atom has probably a high energetic barrier as found in similar reaction mechanisms studied by quantum mechanics.^60−62^ Therefore, it would seem that the stepwise mechanism, which involves an activation of Cys647 by Asp473, is the most probable initial reaction of the catalytic mechanism in PAD2 (Figure 12).

## Conclusions

Five different systems were defined on the basis of the different possible protonation states of the active site residues: Cys647, His471, Asp351, and Asp473. To describe the conformational space as broadly as possible, 10 independent MD runs with a length of 1 μs were performed for each defined system. A total of 50 independent MD runs of 1 μs were carried out to provide a deeper understanding of the structural changes within the PAD2 homodimer complex.

Radial distribution functions of the interactions of Cys647 with solvent water clearly show that for the three systems containing a negatively charged Cys647 (PS-II–PS-IV), two water molecules on average form strong hydrogen bonds with the catalytic cysteine and prevent the latter from attacking the substrate. This demonstrates that Cys647 must be protonated during the initial step of the catalytic reaction.

PS-I and PS-V differ from the protonation state of His471. In PS-I, His471 is neutral, while in PS-V, the imidazole ring is positively charged. The stabilities of the substrate during the MD simulations were very different. For many PS-V trajectories, the substrate lost surface contact with the active site residues. In contrast, the guanidinium fragment of BAEE maintained a stable interaction with the neutral His471 for all PS-I simulations. The results have been emphasized by LIE analysis that shows that the interactions between the substrate and His471 are repulsive for PS-V and attractive for PS-I. We interpret this findings by stating that His471 should be neutral to accommodate a PAD2 substrate and that PS-I represents the only valid protonation state to start the catalytic reaction.

From the PS-I state, several reaction mechanisms can be envisaged. We have used NAC to evaluate the different probabilities of finding conformations that could initiate the catalytic reaction. Overall, we have found frames compatible with all possible mechanisms. Further quantum mechanical or QM/MM works will have to determine the different energetic barriers of every possible mechanisms starting from neutral Cys647 and His471. However, given the presence in the vicinity of Cys647 of an aspartate residue, Asp473, that could play the role of a general base to activate the catalytic cysteine, we hypothesize that the PAD2 catalytic mechanism should be very similar to the proposed reaction mechanism depicted in Figure 12.

## Supporting Information Available

Scripts and figures for the parametrization of the BAEE ligand molecule, RMSD analysis, RDF analysis, surface contact computations, LIE analysis, NAC analysis, RDF analysis between Asp473 and the substrate, and analysis of the distance between Asp351 and the substrate (PDF)
Coordinates of the last frames of all 10 simulations and five systems with the 100 closest water molecules from each active site (ZIP)
