# Supplementary material for: Molecular Mechanism of Protein Arginine Deiminase 2: A Study Involving Multiple Microsecond Long Molecular Dynamics Simulations
Source: Biochemistry. 2022 Jun 23;61(13):1286–97. doi: 10.1021/acs.biochem.2c00158 (PMC9260958; doi:10.1021/acs.biochem.2c00158)
Supplement: Supplementary file 1 — bi2c00158_si_001.pdf [file bi2c00158_si_001.pdf]

Supporting Information: Molecular Mechanism of Protein Arginine Deiminase 2: A Study Involving Multiple Microsecond Long Molecular Dynamics Simulations

Erdem Cicek,[‡] Gerald Monard,[*,§] and Fethiye Aylin Sungur[*,‡]

‡*Istanbul Technical University, Informatics Institute, Computational Science and Engineering, TR-34469 Istanbul, Turkey*

§*Université de Lorraine, CNRS, LPCT, F-54000 Nancy, France*

E-mail: gerald.monard@univ-lorraine.fr; aylin.sungur@itu.edu.tr

Parametrization Of Ligand Molecule

Scripts for Parametrization

```
# Create Gaussian input file
$ antechamber -i BEN-NME.pdb -fi pdb -o BEN-NME.com -fo gcrt -gv 1
    -ge BEN-NME.gesp -gn '%NProcShared=8' -gm "%Mem=8Gb" -rn BEN -at amber
$ antechamber -i ACE-ETH.pdb -fi pdb -o ACE-ETH.com -fo gcrt -gv 1
    -ge ACE-ETH.gesp -gn '%NProcShared=8' -gm "%Mem=8Gb" -rn ETH -at amber

# Create antechamber file
$ antechamber -i BEN-NME.pdb  -fi pdb -o BEN-NME.ac  -fo ac -nc  0  -at amber  -pf y
$ antechamber -i ACE-ETH.pdb  -fi pdb -o ACE-ETH.ac  -fo ac -nc  0  -at amber  -pf y

# Generate ESP files from Gaussian outputs
$ espgen -i BEN-NME.gesp -o BEN-NME.esp
$ espgen -i ACE-ETH.gesp -o ACE-ETH.esp

# residuegen
$ cat > BEN-NME.residuegen << END
INPUT_FILE        BEN-NME.ac
CONF_NUM          1
ESP_FILE          BEN-NME.esp
SEP_BOND          C N
NET_CHARGE        0
PREP_FILE         BEN.prep
RESIDUE_FILE_NAME BEN.res
RESIDUE_SYMBOL    BEN
END

$ residuegen BEN-NME.residuegen

$ cat > ACE-ETH.residuegen << END
INPUT_FILE        ACE-ETH.ac
CONF_NUM          1
ESP_FILE          ACE-ETH.esp
SEP_BOND          OS C
NET_CHARGE        0
PREP_FILE         ETH.prep
RESIDUE_FILE_NAME ETH.res
RESIDUE_SYMBOL    ETH
```

```
END

$ residuegen ACE-ETH.residuegen

# respgen
$ respgen -i BEN-NME.ac -o BEN-NME.respin1 -f resp1
$ respgen -i ACE-ETH.ac -o ACE-ETH.respin1 -f resp1

# create GROUP fragment  containing CAP atoms
$ cat > BEN-NME.grp << END
GROUP  6  0.000
ATOM     14  N
ATOM     15  H
ATOM     16  CH1
ATOM     17  HH11
ATOM     18  HH12
ATOM     19  HH13
END

$ cat > ACE-ETH.grp << END
GROUP  6  0.000
ATOM      9  C
ATOM     10  O
ATOM     11  CH
ATOM     12  HH1
ATOM     13  HH2
ATOM     14  HH3
END

$ respgen -i BEN-NME.ac -o BEN-NME.respin2 -f resp2 -a BEN-NME.grp
$ respgen -i ACE-ETH.ac -o ACE-ETH.respin2 -f resp2 -a ACE-ETH.grp

# post-editing: change ACE-ETH.respin2
$ cat > ACE-ETH.respin2 << END
Resp charges for organic molecule

 &cntrl

 nmol = 1,
 ihfree = 1,
 ioutopt = 1,
```

```
 iqopt = 2,
 qwt =  0.00100,

 &end
    1.0
Resp charges for organic molecule
    0   14
    1    0
    6    0
    1    1
    1    1
    6    0
    8    0
    8    0
    6    0
    1    0
    1    9
    6  -99
    1    0
    1   12
    1   12
    6   0.000
    1    1    1    2    1    3    1    4    1    5    1    6
END

# run RESP (two stages)
$ resp -O -i BEN-NME.respin1 -o BEN-NME.respout1 -e BEN-NME.esp \
    -t BEN-NME.stage1
$ resp -O -i BEN-NME.respin2 -o BEN-NME.respout2 -e BEN-NME.esp \
    -q BEN-NME.stage1 -t BEN-NME.stage2
$ resp -O -i ACE-ETH.respin1 -o ACE-ETH.respout1 -e ACE-ETH.esp \
    -t ACE-ETH.stage1
$ resp -O -i ACE-ETH.respin2 -o ACE-ETH.respout2 -e ACE-ETH.esp \
    -q ACE-ETH.stage1 -t ACE-ETH.stage2

# read charges and output Antechamber AC
$ antechamber -i BEN-NME.ac -fi ac -o BEN-NME-resp.ac -fo ac -c rc -cf BEN-NME.stage2
    -pf y -at amber
$ antechamber -i ACE-ETH.ac -fi ac -o ACE-ETH-resp.ac -fo ac -c rc -cf ACE-ETH.stage2
    -pf y -at amber
# create prep file
```

```
$ cat > BEN-NME.chain << END
TAIL_NAME C
MAIN_CHAIN CA
MAIN_CHAIN CB1
MAIN_CHAIN CB2
MAIN_CHAIN CG1
MAIN_CHAIN CG2
MAIN_CHAIN CD
OMIT_NAME N
OMIT_NAME H
OMIT_NAME CH1
OMIT_NAME HH11
OMIT_NAME HH12
OMIT_NAME HH13
POST_TAIL_TYPE N
CHARGE 0.0
END

$ cat > ACE-ETH.chain << END
HEAD_NAME OS
MAIN_CHAIN CA
MAIN_CHAIN CB
OMIT_NAME C
OMIT_NAME O
OMIT_NAME CH
OMIT_NAME HH1
OMIT_NAME HH2
OMIT_NAME HH3
PRE_HEAD_TYPE C
CHARGE 0.0
END

$ prepgen -i BEN-NME-resp.ac -o BEN.prep -rn BEN -rf BEN.res \
    -m BEN-NME.chain -f int
$ prepgen -i ACE-ETH-resp.ac -o ETH.prep -rn ETH -rf ACE.res \
    -m ACE-ETH.chain -f int

$ cat > BEN-ARG-ETH.leapin << END
source leaprc.protein.ff14SB
loadamberprep BEN.prep
loadamberprep ETH.prep
```

```
loadamberparams BEN-ARG-ETH.frcmod
mol = sequence { BEN ARG ETH }
saveamberparm mol BEN-ARG-ETH.top BEN-ARG-ETH.crd
quit
END
```

```
$ ambpdb -p BEN-ARG-ETH.top < BEN-ARG-ETH.crd > BEN-ARG-ETH.pdb
```

RMSD Analysis of All Systems

Scripts for Data Preparation

```
#!/bin/bash
function trajin()
{
for j in {00..09};
    do
      cat > ${fileName}-${j}.in << END
parm ${dir}/${j}/md.top
trajin ${dir}/${j}/sampling-???.nc

rms all :1-1307@CA first out ${fileName}-${j}.dat time 0.04
rms dimer :1-645,655-1299@CA first out ${fileName}-${j}.dat time 0.04

rms chainA :1-645@CA first out ${fileName}-${j}.dat time 0.04
rms chainB :655-1299@CA first out ${fileName}-${j}.dat time 0.04
rms caions @%Ca2+ first out ${fileName}-${j}.dat time 0.04

rms activeA :335,388,455,456,457,626,627@CA first out ${fileName}-${j}.dat mass time 0.04
rms subsA :652,653,654@CA first nofit out ${fileName}-${j}.dat mass time 0.04
rms active-subsA :335,388,455,456,457,626,627,652,653,654@CA first out ${fileName}-${j}.dat mass time 0.04

rms activeB :989,1042,1109,1110,1111,1280,1281@CA first out ${fileName}-${j}.dat mass time 0.04
rms subsB :1306,1307,1308@CA first nofit out ${fileName}-${j}.dat mass time 0.04
rms active-subsB :989,1042,1109,1110,1111,1280,1281,1306,1307,1308@CA first out ${fileName}-${j}.dat mass time 0.04

run
END
      cpptraj.OMP -i ${fileName}-${j}.in
done
}

rootPath=/mnt/beegfs/ese86/shared/Erdem

fileName=ps1-rmsd dir=${rootPath}/CYS-HIE-ASP trajin
fileName=ps2-rmsd dir=${rootPath}/CYM-HIE-ASH trajin
fileName=ps3-rmsd dir=${rootPath}/CYM-HIP-ASP trajin
fileName=ps4-rmsd dir=${rootPath}/CYM-HIP-ASH trajin
```

```
fileName=ps5-rmsd dir=${rootPath}/CYS-HIP-ASP trajin
```

RMSD Plots of PS-I System

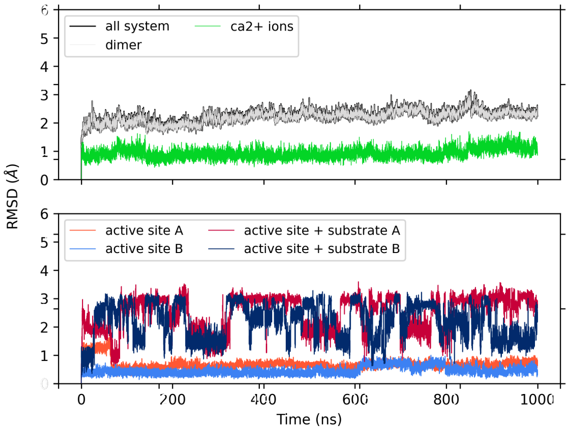

(a) Sim-00

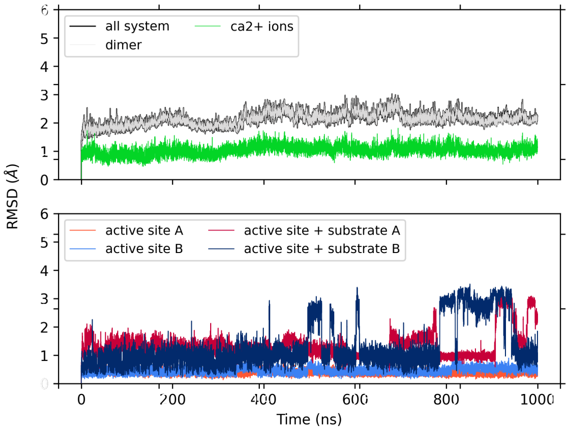

(b) Sim-01

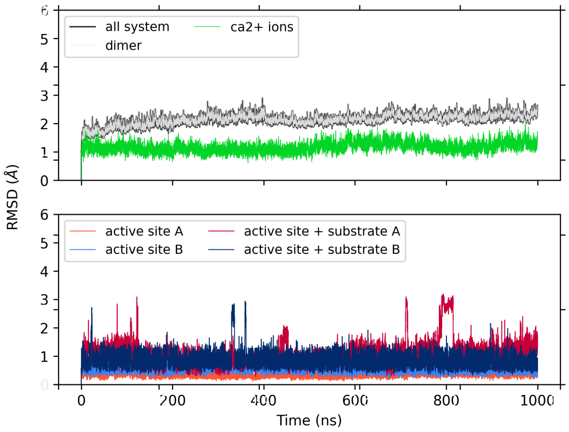

(c) Sim-02

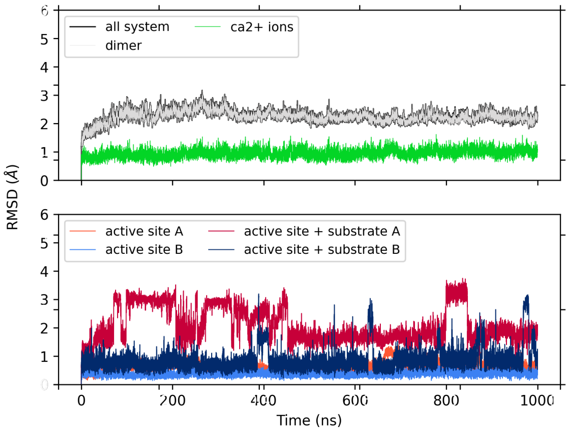

(d) Sim-03

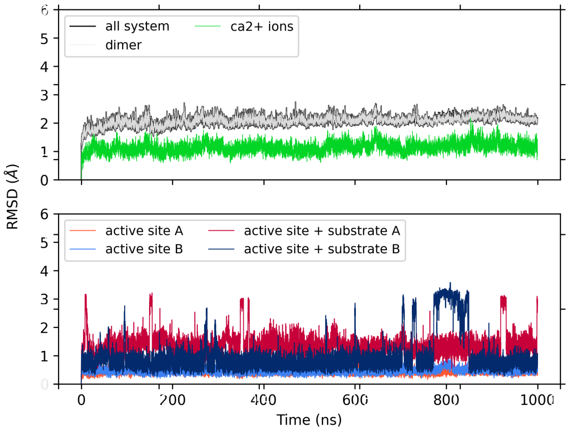

(e) Sim-04

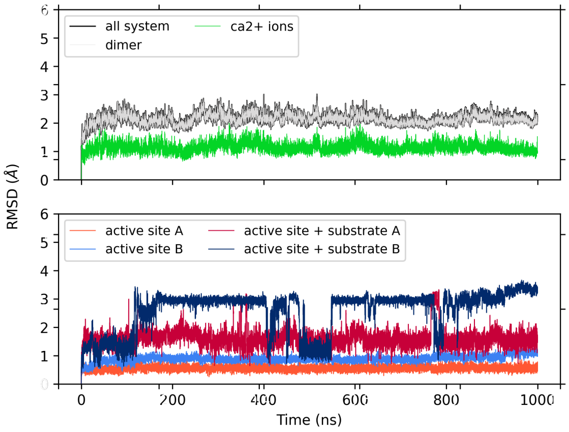

(f) Sim-05

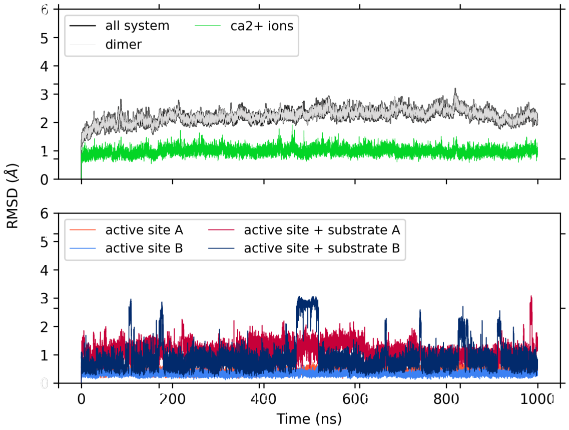

(g) Sim-06

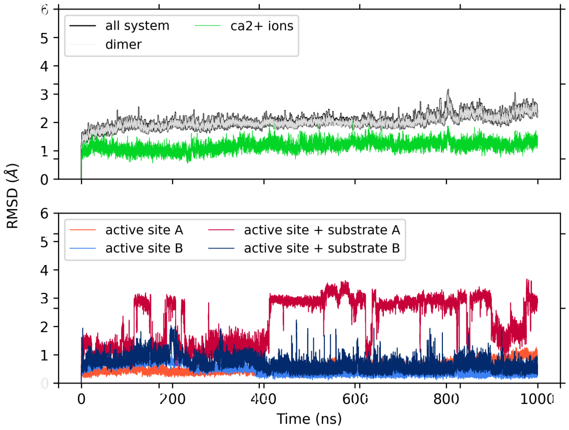

(h) Sim-07

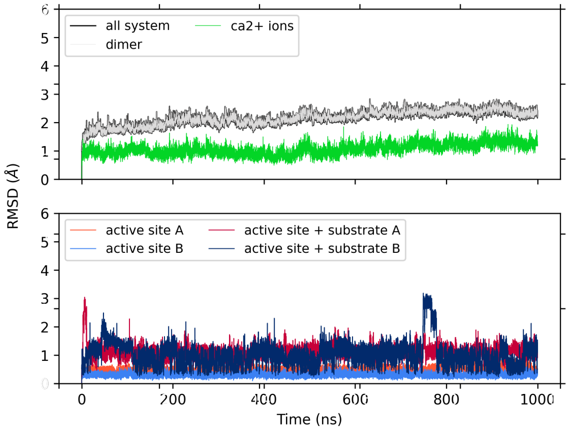

(i) Sim-08

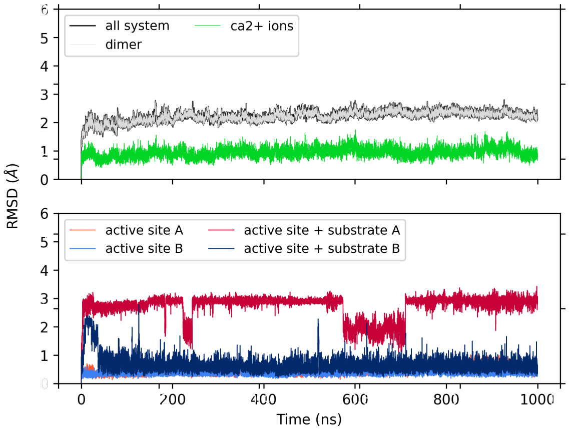

(j) Sim-09

Figure S1: Root mean square deviation of the protein backbone from the first structure during the 10 PS-I simulations.

RMSD Plots of PS-II System

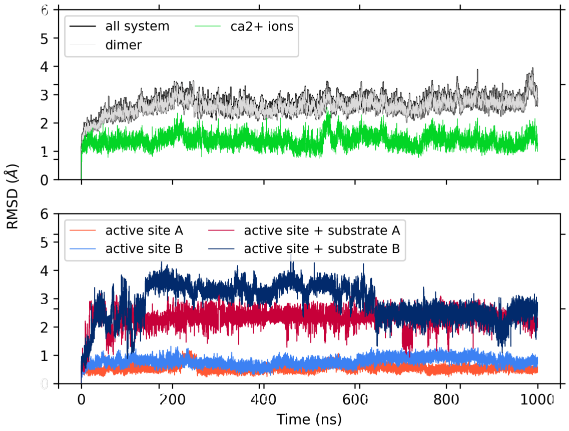

(a) Sim-00

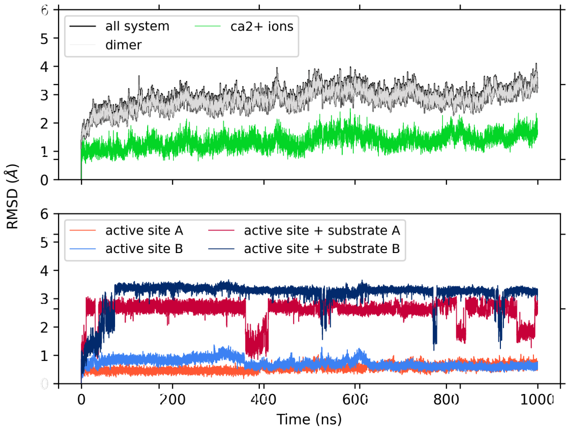

(b) Sim-01

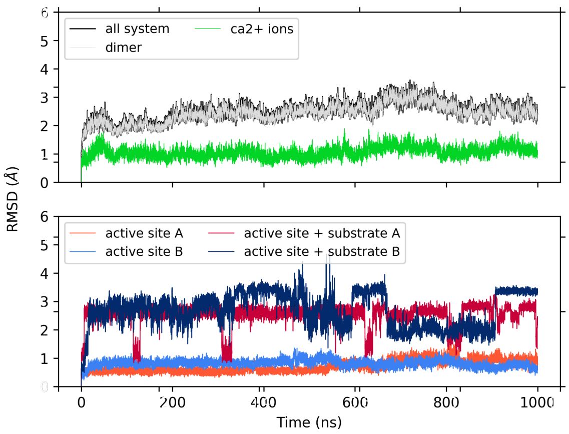

(c) Sim-02

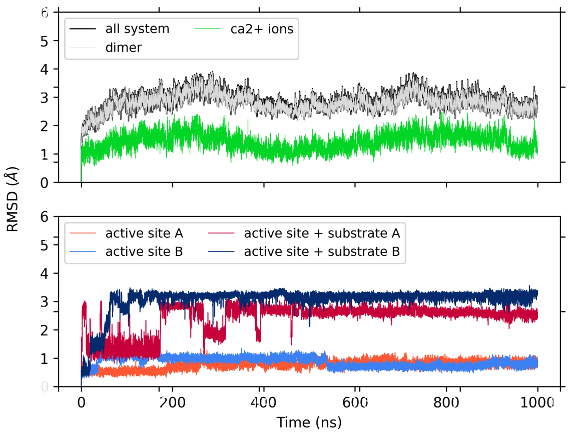

(d) Sim-03

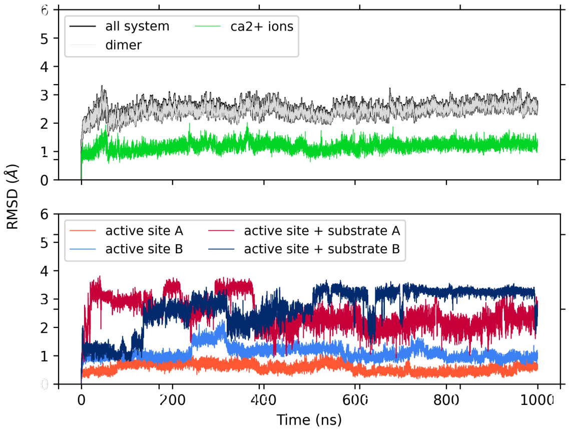

(e) Sim-04

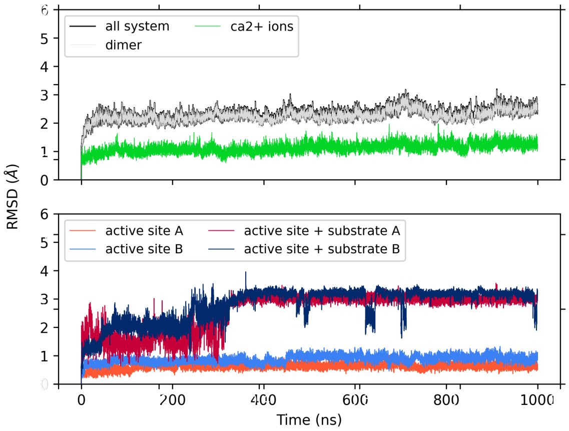

(f) Sim-05

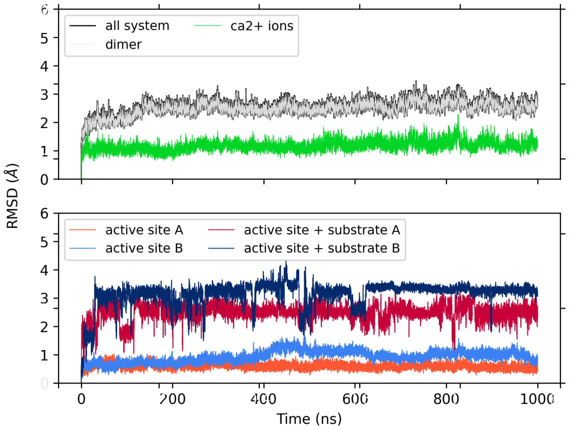

(g) Sim-06

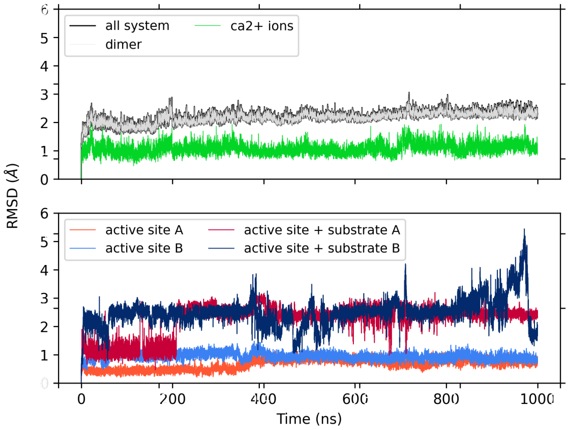

(h) Sim-07

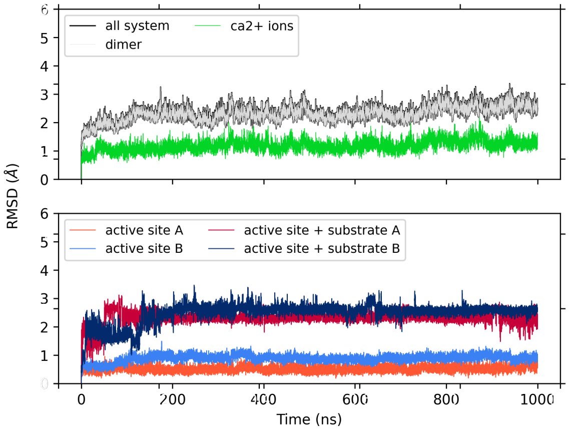

(i) Sim-08

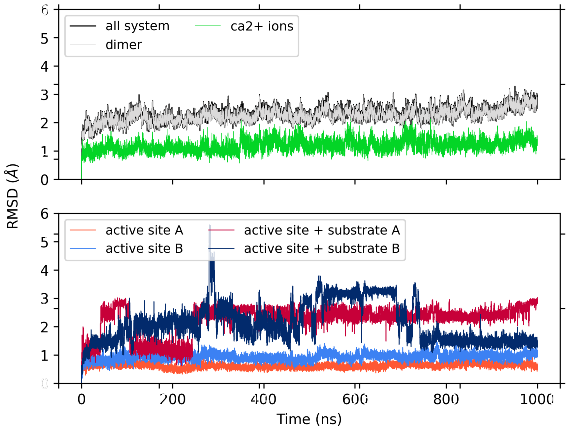

(j) Sim-09

Figure S2: Root mean square deviation of the protein backbone from the first structure during the 10 PS-II simulations.

RMSD Plots of PS-III System

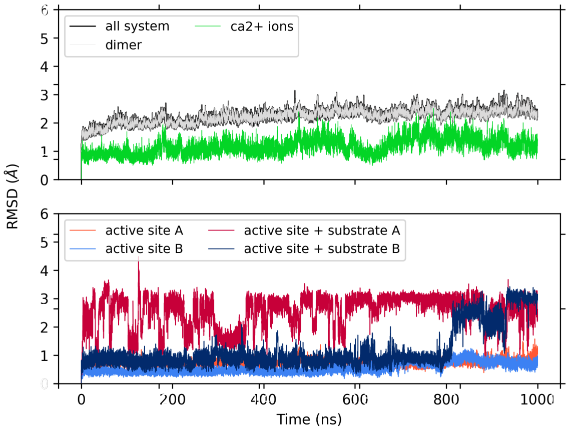

(a) Sim-00

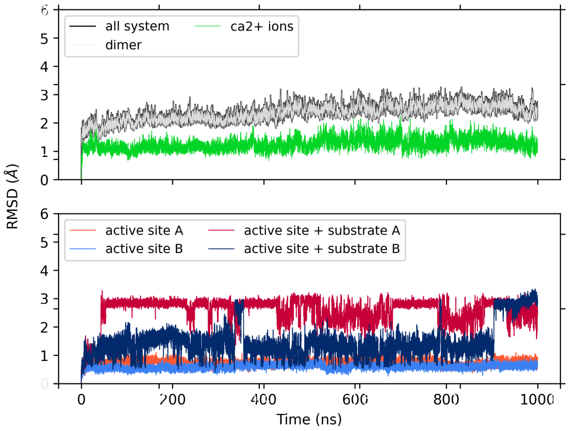

(b) Sim-01

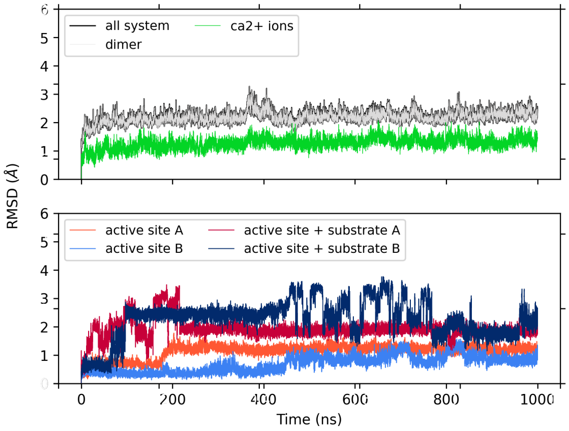

(c) Sim-02

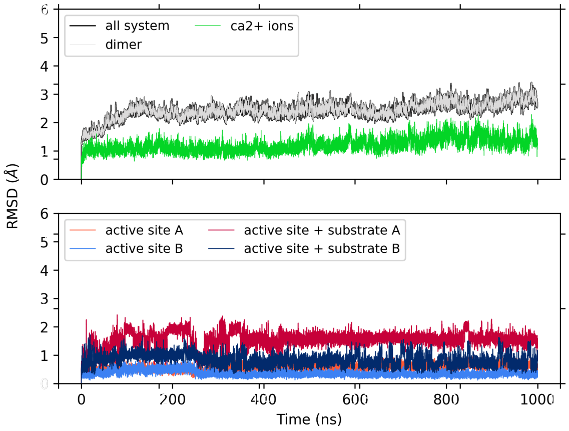

(d) Sim-03

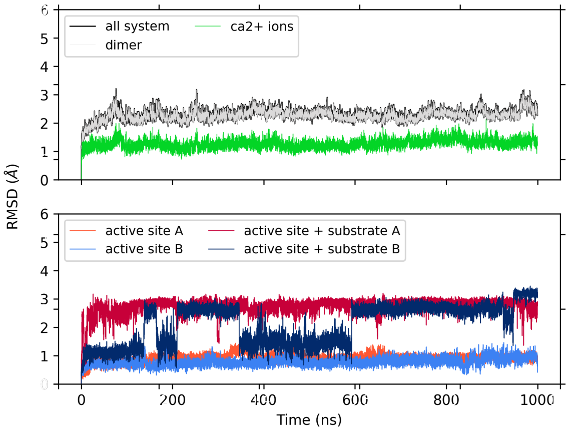

(e) Sim-04

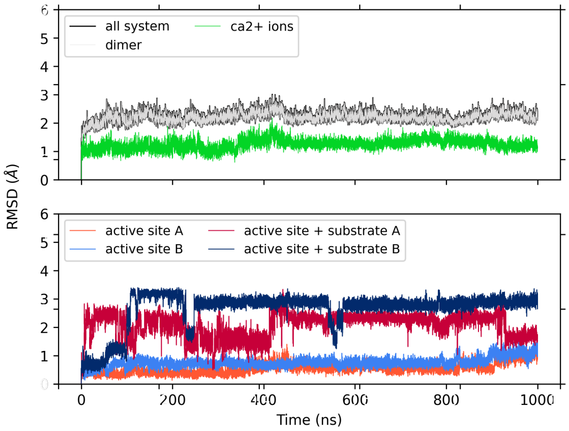

(f) Sim-05

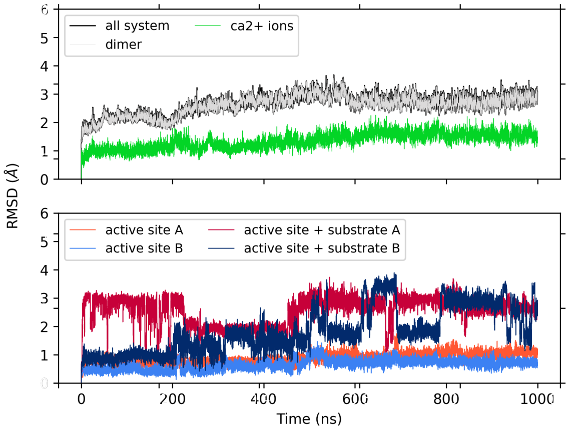

(g) Sim-06

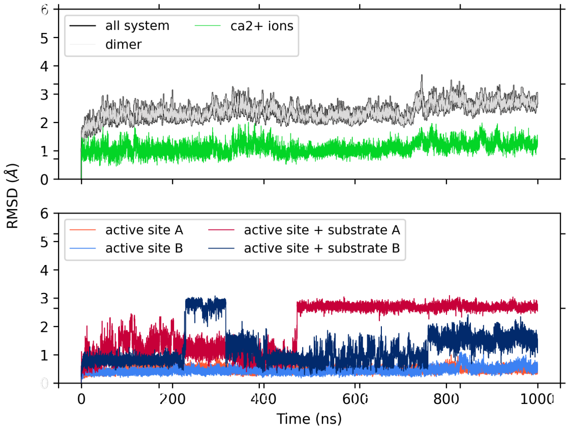

(h) Sim-07

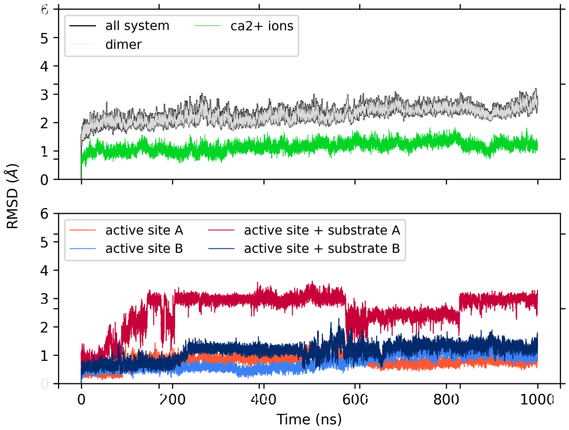

(i) Sim-08

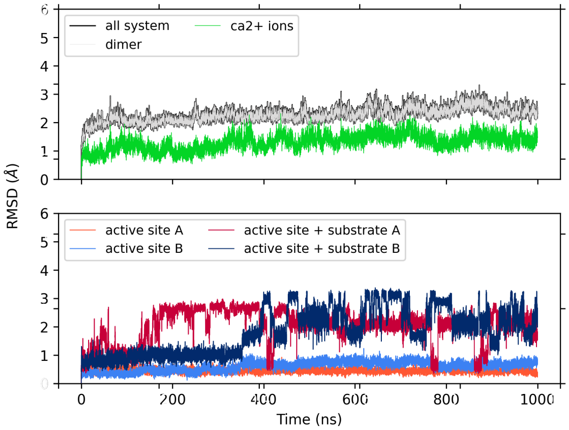

(j) Sim-09

Figure S3: Root mean square deviation of the protein backbone from the first structure during the 10 PS-III simulations.

RMSD Plots of PS-IV System

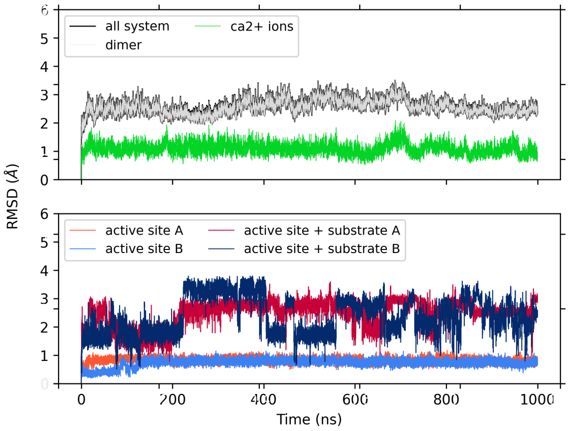

(a) Sim-00

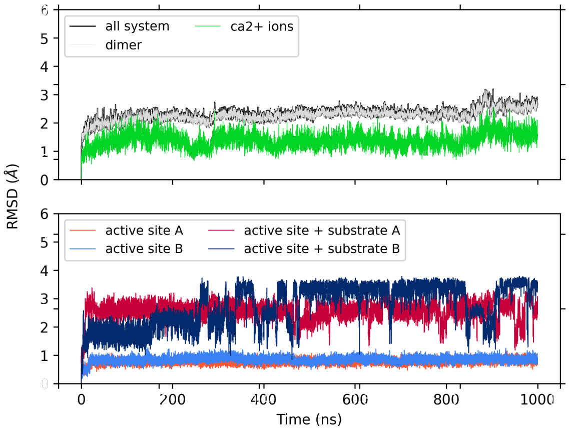

(b) Sim-01

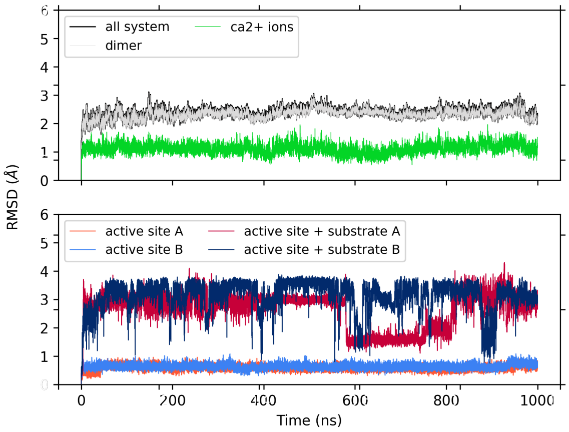

(c) Sim-02

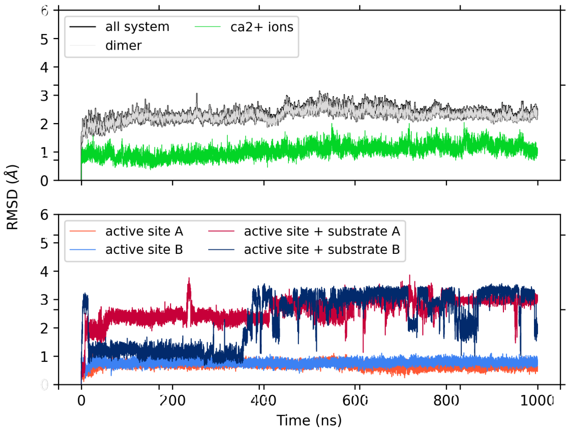

(d) Sim-03

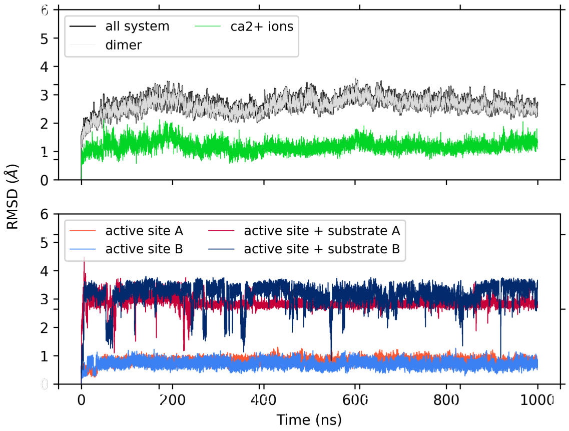

(e) Sim-04

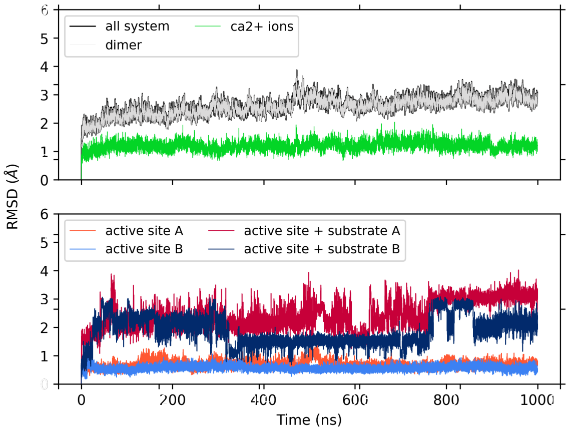

(f) Sim-05

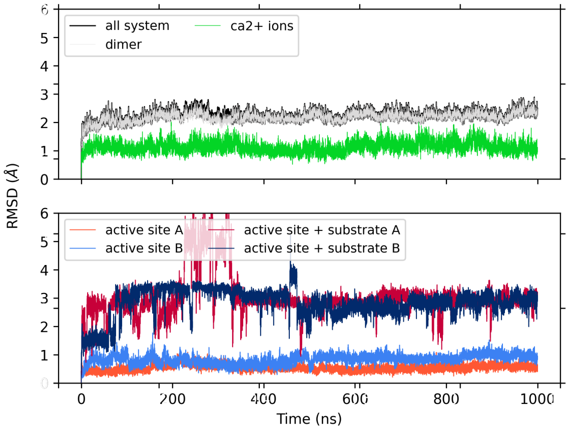

(g) Sim-06

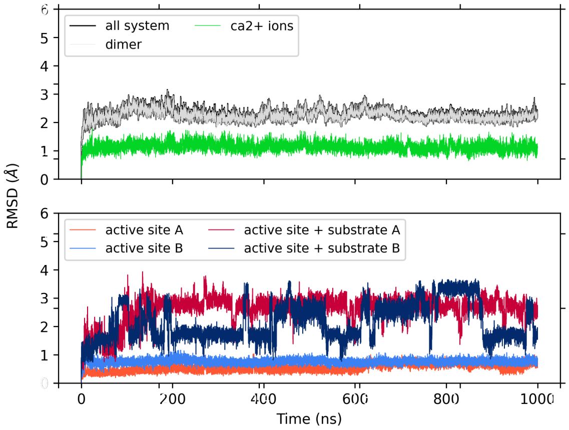

(h) Sim-07

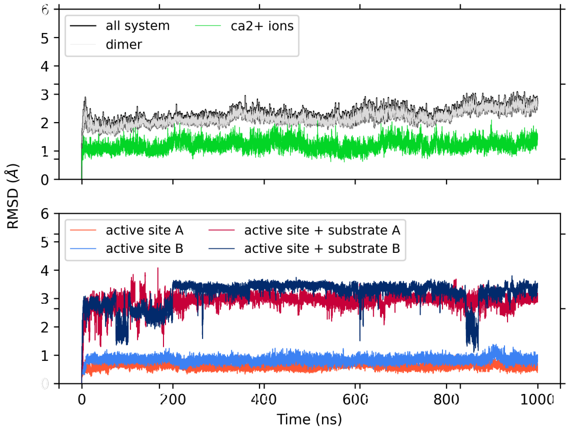

(i) Sim-08

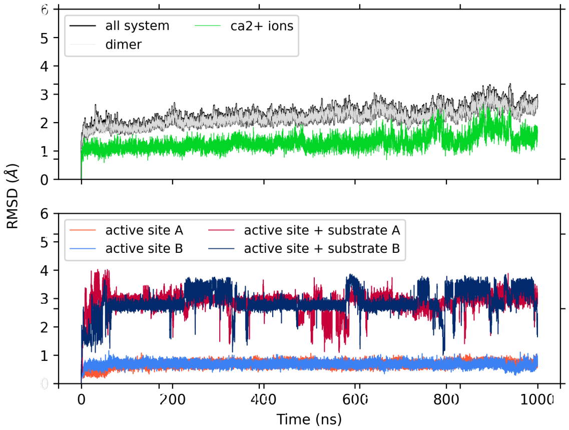

(j) Sim-09

Figure S4: Root mean square deviation of the protein backbone from the first structure during the 10 PS-IV simulations.

RMSD Plots of PS-V System

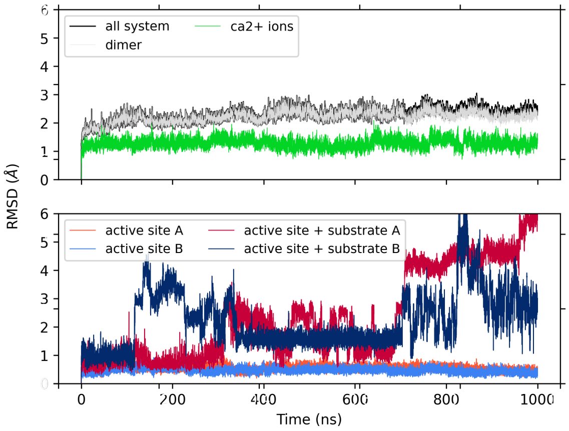

(a) Sim-00

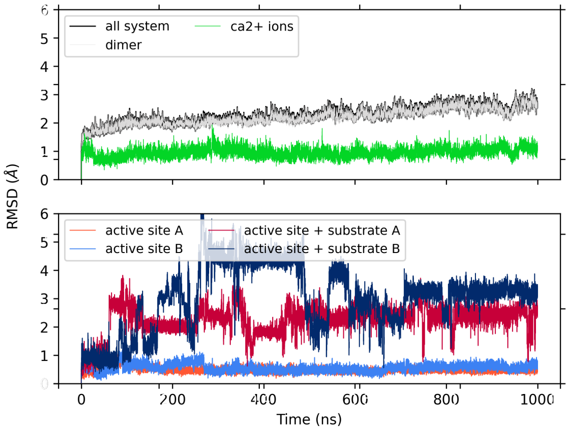

(b) Sim-01

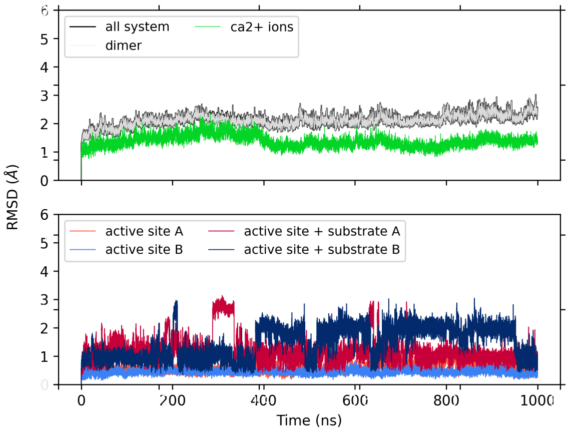

(c) Sim-02

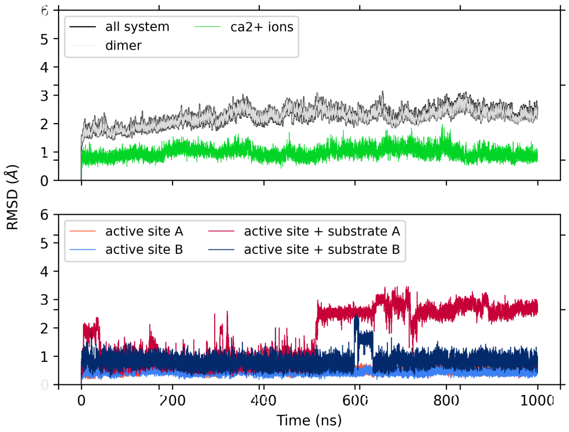

(d) Sim-03

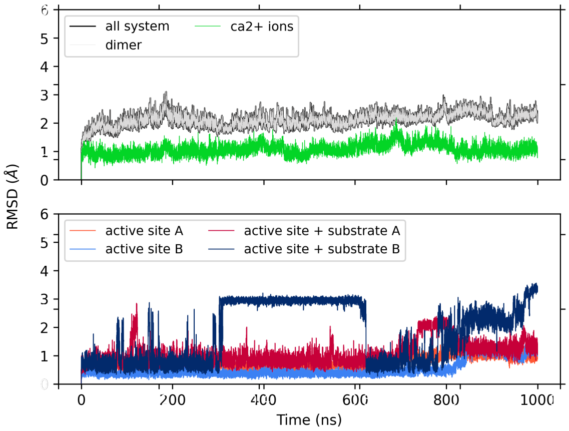

(e) Sim-04

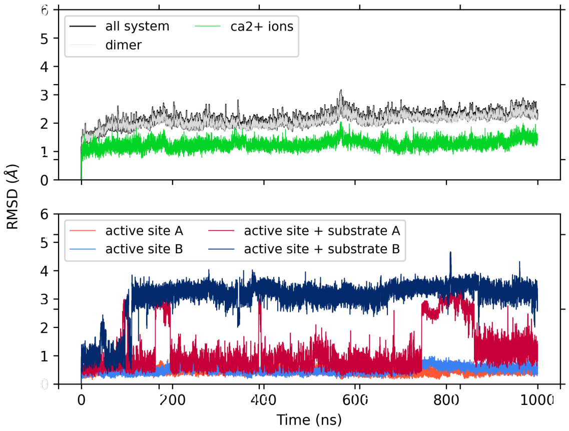

(f) Sim-05

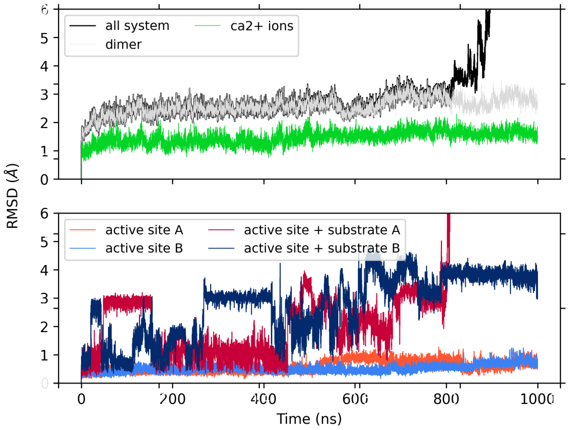

(g) Sim-06

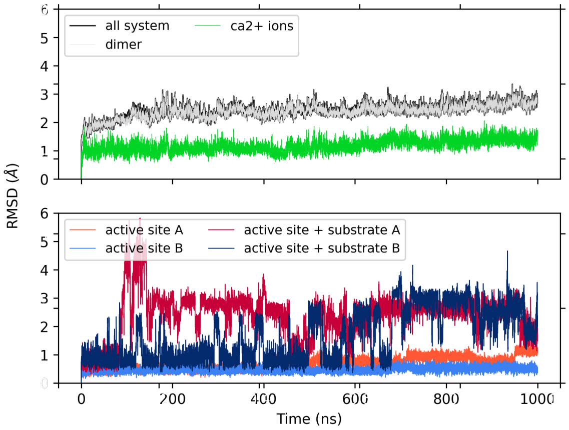

(h) Sim-07

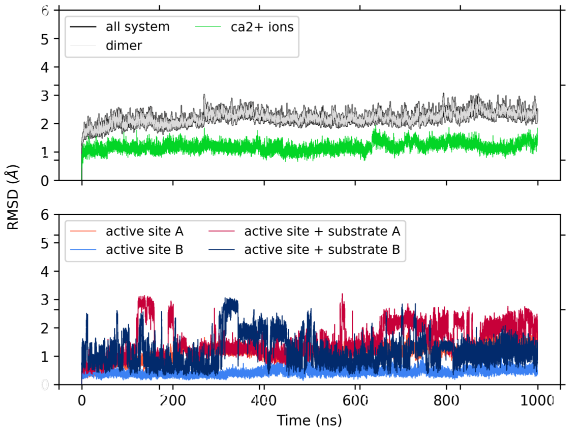

(i) Sim-08

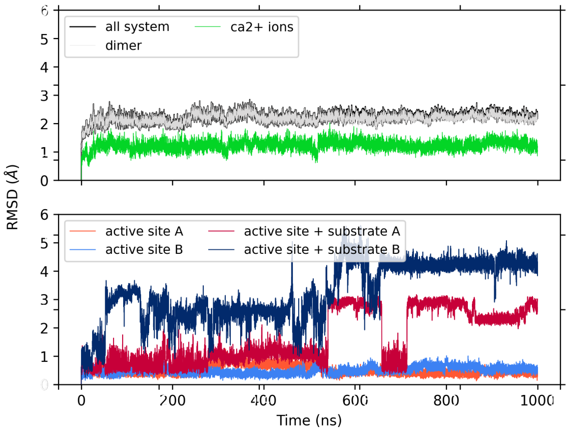

(j) Sim-09

Figure S5: Root mean square deviation of the protein backbone from the first structure during the 10 PS-V simulations.

Radial Distribution Function Analysis of All Systems

Scripts for Data Preparation

```bash
#!/bin/bash

system='CYS-HIE-ASP CYM-HIE-ASH CYM-HIP-ASP CYM-HIP-ASH CYS-HIP-ASP'

function trajinA()
{
cpptraj.OMP << END
parm ${dir}/${j}/md.top
trajin ${dir}/${j}/sampling-???.nc

radial ${fileName}-1.dat  0.1 100.0 :WAT@H1,H2                  :627@SG      volume \
       intrdf ${fileName}-1.dat  rawrdf ${fileName}-1.dat
radial ${fileName}-2.dat  0.1 100.0 :653@HE,HH11,HH12,HH21,HH22 :627@SG      volume \
       intrdf ${fileName}-2.dat  rawrdf ${fileName}-2.dat
radial ${fileName}-3.dat  0.1 100.0 :653@HE,HH11,HH12,HH21,HH22 :457@OD1,OD2 volume \
       intrdf ${fileName}-3.dat  rawrdf ${fileName}-3.dat
radial ${fileName}-4a.dat 0.1 100.0 :653@NE,NH1,NH2             :455@ND1     volume \
       intrdf ${fileName}-4a.dat rawrdf ${fileName}-4a.dat
radial ${fileName}-4b.dat 0.1 100.0 :653@NE,NH1,NH2             :455@HD1     volume \
       intrdf ${fileName}-4b.dat rawrdf ${fileName}-4b.dat
radial ${fileName}-5a.dat 0.1 100.0 :457@OD1,OD2                :627@SG      volume \
       intrdf ${fileName}-5a.dat rawrdf ${fileName}-5a.dat
radial ${fileName}-5b.dat 0.1 100.0 :457@OD1,OD2                :627@HG      volume \
       intrdf ${fileName}-5b.dat rawrdf ${fileName}-5b.dat
radial ${fileName}-6.dat  0.1 100.0 :WAT@O                      :627@HG      volume \
       intrdf ${fileName}-6.dat  rawrdf ${fileName}-6.dat
radial ${fileName}-7a.dat 0.1 100.0 :WAT@O                      :457@OD1,OD2 volume \
       intrdf ${fileName}-7a.dat rawrdf ${fileName}-7a.dat
radial ${fileName}-7b.dat 0.1 100.0 :WAT@H1,H2                  :457@OD1,OD2 volume \
       intrdf ${fileName}-7b.dat rawrdf ${fileName}-7b.dat
END
}

function trajinB()
{
cpptraj.OMP << END
```

```
parm ${dir}/${j}/md.top
trajin ${dir}/${j}/sampling-???.nc

radial ${fileName}-1.dat  0.1 100.0 :WAT@H1,H2                   :1281@SG      volume \
       intrdf ${fileName}-1.dat  rawrdf ${fileName}-1.dat
radial ${fileName}-2.dat  0.1 100.0 :1307@HE,HH11,HH12,HH21,HH22 :1281@SG      volume \
       intrdf ${fileName}-2.dat  rawrdf ${fileName}-2.dat
radial ${fileName}-3.dat  0.1 100.0 :1307@HE,HH11,HH12,HH21,HH22 :1111@OD1,OD2 volume \
       intrdf ${fileName}-3.dat  rawrdf ${fileName}-3.dat
radial ${fileName}-4a.dat 0.1 100.0 :1307@NE,NH1,NH2             :1109@ND1     volume \
       intrdf ${fileName}-4a.dat rawrdf ${fileName}-4a.dat
radial ${fileName}-4b.dat 0.1 100.0 :1307@NE,NH1,NH2             :1109@HD1     volume \
       intrdf ${fileName}-4b.dat rawrdf ${fileName}-4b.dat
radial ${fileName}-5a.dat 0.1 100.0 :1111@OD1,OD2                :1281@SG      volume \
       intrdf ${fileName}-5a.dat rawrdf ${fileName}-5a.dat
radial ${fileName}-5b.dat 0.1 100.0 :1111@OD1,OD2                :1281@HG      volume \
       intrdf ${fileName}-5b.dat rawrdf ${fileName}-5b.dat
radial ${fileName}-6.dat  0.1 100.0 :WAT@O                       :1281@HG      volume \
       intrdf ${fileName}-6.dat  rawrdf ${fileName}-6.dat
radial ${fileName}-7a.dat 0.1 100.0 :WAT@O                       :1111@OD1,OD2 volume \
       intrdf ${fileName}-7a.dat rawrdf ${fileName}-7a.dat
radial ${fileName}-7b.dat 0.1 100.0 :WAT@H1,H2                   :1111@OD1,OD2 volume \
       intrdf ${fileName}-7b.dat rawrdf ${fileName}-7b.dat
END
}

fileName=radialA-PS1 dir=CYS-HIE-ASP trajin
fileName=radialA-PS2 dir=CYM-HIE-ASH trajin
fileName=radialA-PS3 dir=CYM-HIP-ASP trajin
fileName=radialA-PS4 dir=CYM-HIP-ASH trajin
fileName=radialA-PS5 dir=CYS-HIP-ASP trajin

fileName=radialB-PS1 dir=CYS-HIE-ASP trajin
fileName=radialB-PS2 dir=CYM-HIE-ASH trajin
fileName=radialB-PS3 dir=CYM-HIP-ASP trajin
fileName=radialB-PS4 dir=CYM-HIP-ASH trajin
fileName=radialB-PS5 dir=CYS-HIP-ASP trajin
```

RDF Plots

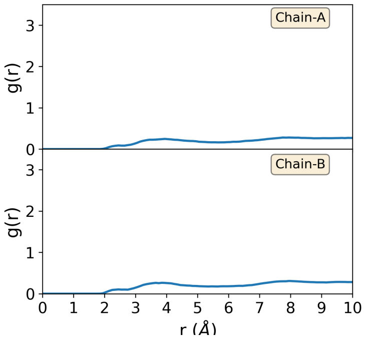

(a) PS-I

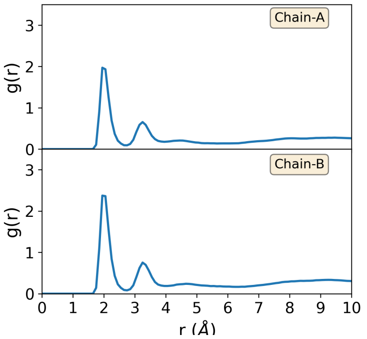

(b) PS-II

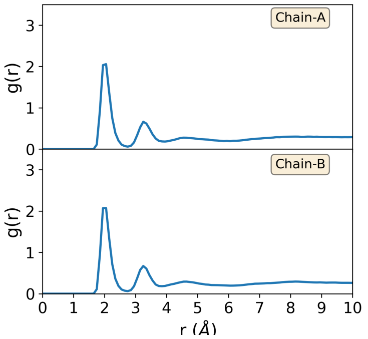

(c) PS-III

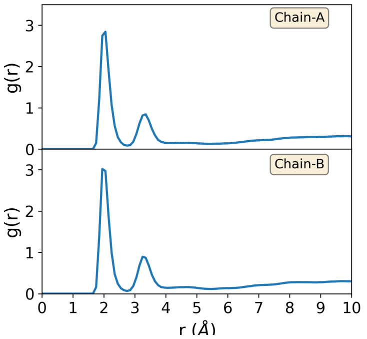

(d) PS-IV

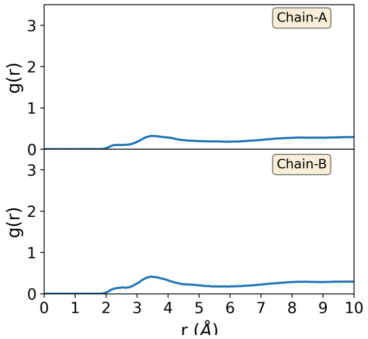

(e) PS-V

Figure S6: Radial Distribution Function analysis of all Systems

Surface Contact Analysis for All Systems

Scripts for Data Preparation

```
#!/bin/bash

function trajin()
{
for j in {00..09}
    do
        cat > ${fileName}-${j}.in << END
 parm ${dir}/${j}/md.top
 trajin ${dir}/${j}/sampling-???.nc

 autoimage :260

 surf allA :627,457,455,335,653 out ${fileName}-A-$j.dat solutemask :627,457,455,335,653
 surf activesiteA :627,457,455,335 out ${fileName}-A-$j.dat solutemask :627,457,455,335
 surf ligandA :653 out ${fileName}-A-$j.dat solutemask :653
 surf allB :1281,1111,1109,989,1307 out ${fileName}-B-$j.dat solutemask :1281,1111,1109,989,1307
 surf activesiteB :1281,1111,1109,989 out ${fileName}-B-$j.dat solutemask :1281,1111,1109,989
 surf ligandB :1307 out ${fileName}-B-$j.dat solutemask :1307
 run
END
        cpptraj.OMP -i ${fileName}.in
done
}

rootPath=/mnt/beegfs/ese86/shared/Erdem

fileName=contact-PS1 dir=${rootPath}/CYS-HIE-ASP trajin
fileName=contact-PS2 dir=${rootPath}/CYM-HIE-ASH trajin
fileName=contact-PS3 dir=${rootPath}/CYM-HIP-ASP trajin
fileName=contact-PS4 dir=${rootPath}/CYM-HIP-ASH trajin
fileName=contact-PS5 dir=${rootPath}/CYS-HIP-ASP trajin
```

Surface Contact Heatmap for PS-II, PS-III and PS-IV Systems

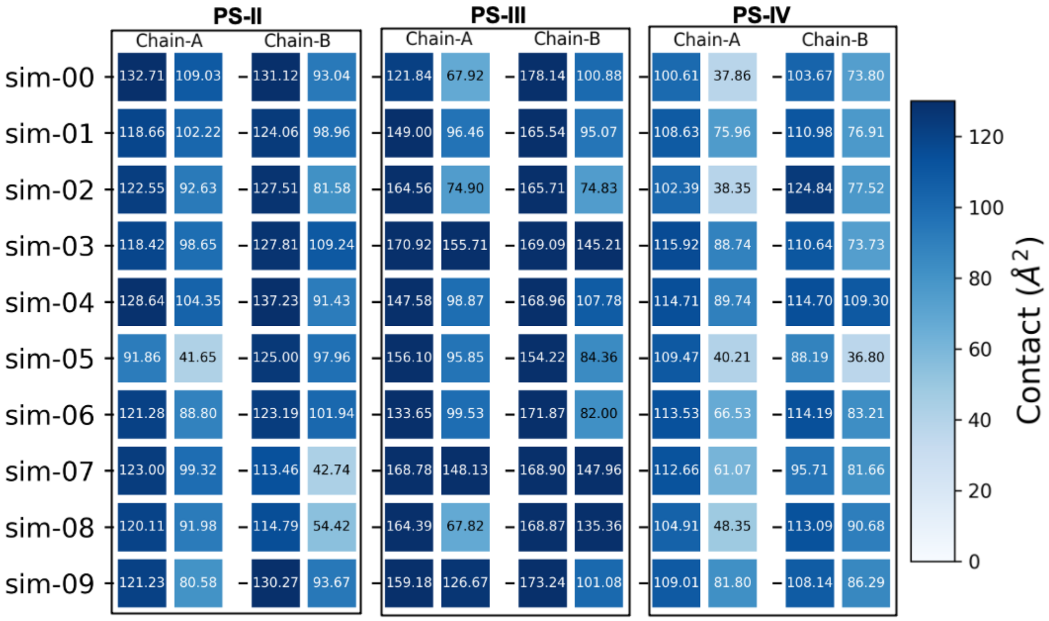

Figure S7: Surface contact area, in Å^2, between the BAEE substrate and the active site residues for both monomer chains (A and B) of the PS-II (left), PS-III (middle) and PS-IV (right) systems. For each chain, the left column represents the maximum surface contact area, the right column represents the surface contact area of the last molecular dynamics frame.

Surface Contact Plots of PS-I System

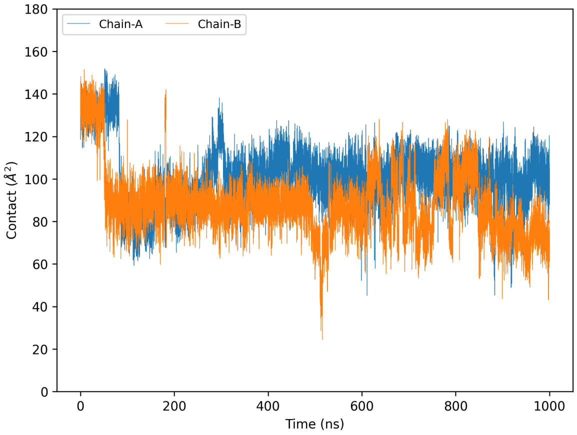

(a) Sim-00

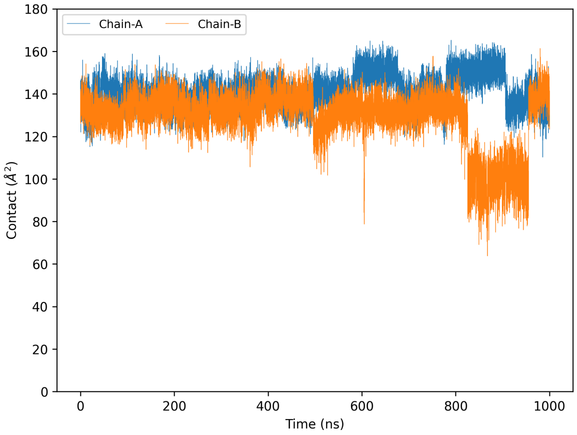

(b) Sim-01

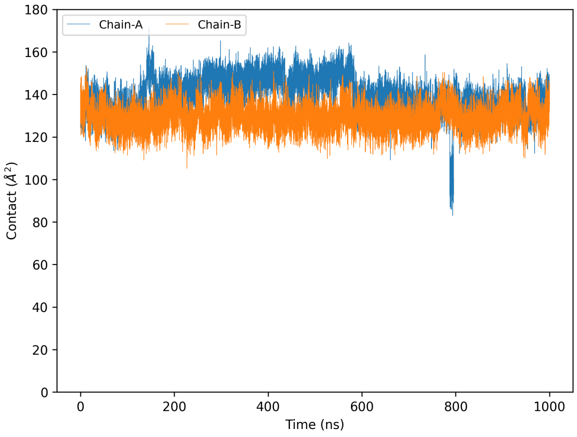

(c) Sim-02

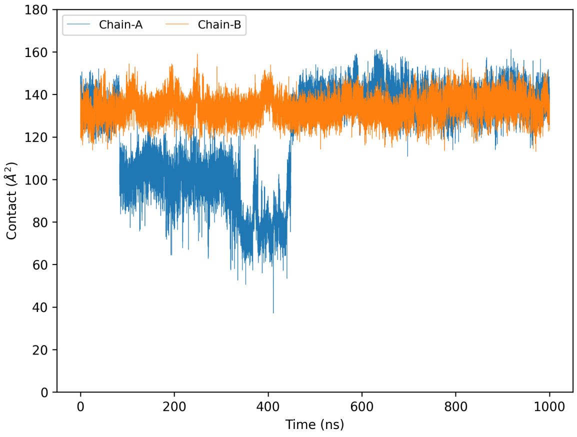

(d) Sim-03

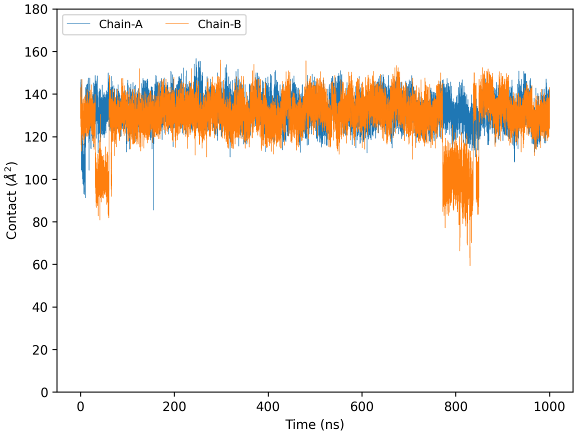

(e) Sim-04

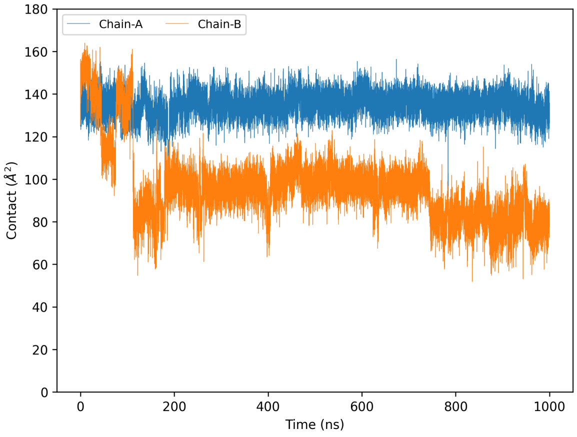

(f) Sim-05

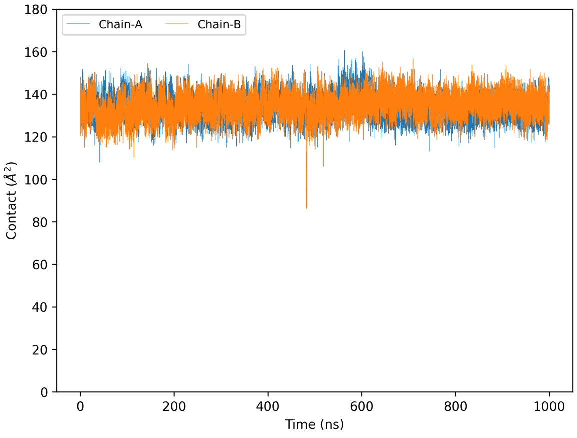

(g) Sim-06

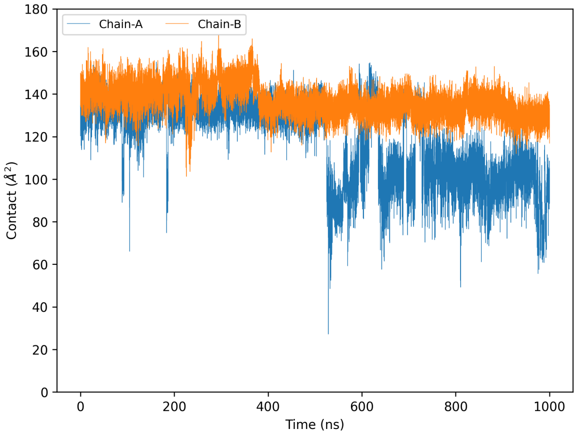

(h) Sim-07

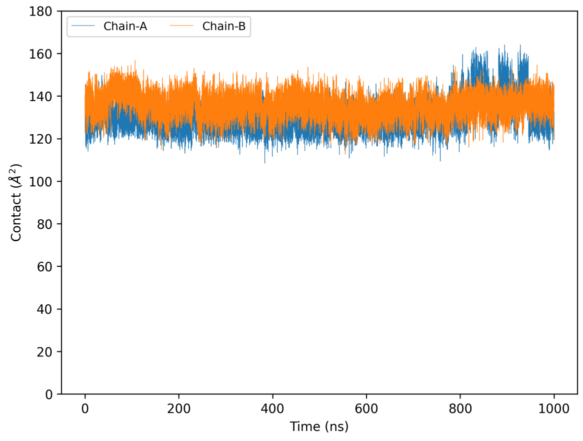

(i) Sim-08

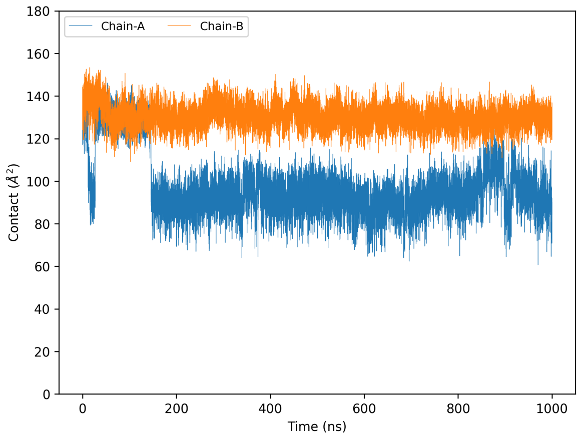

(j) Sim-09

Figure S8: Surface Contact analysis between ligand and active site residues during the 10 PS-I simulations.

Surface Contact Plots of PS-V System

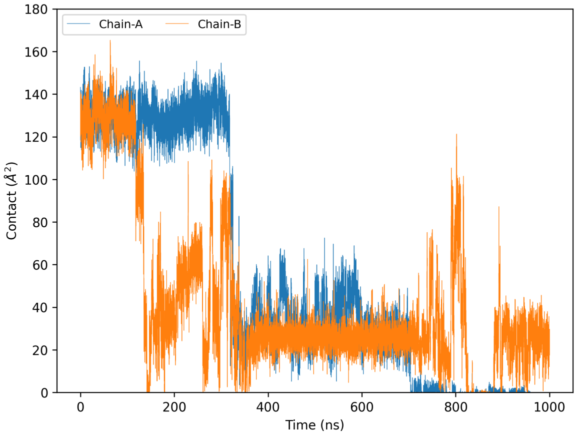

(a) Sim-00

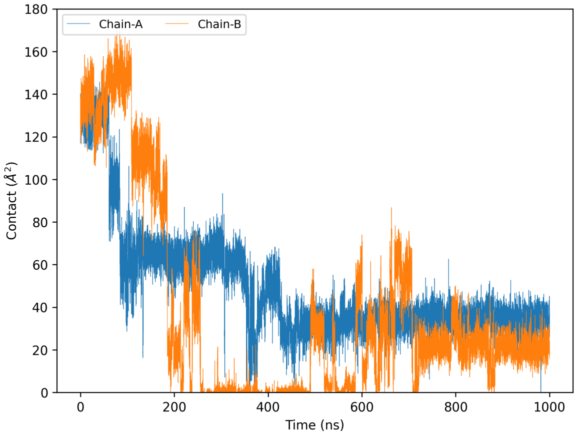

(b) Sim-01

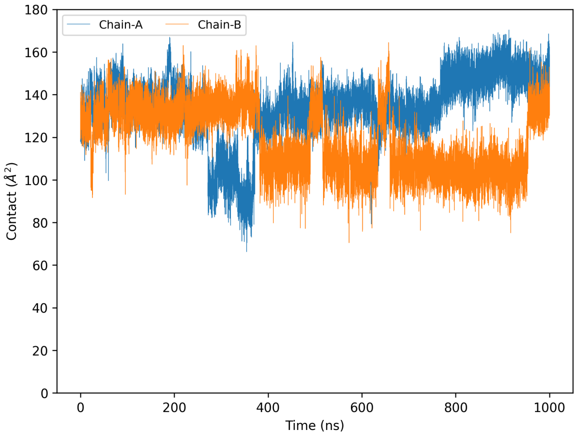

(c) Sim-02

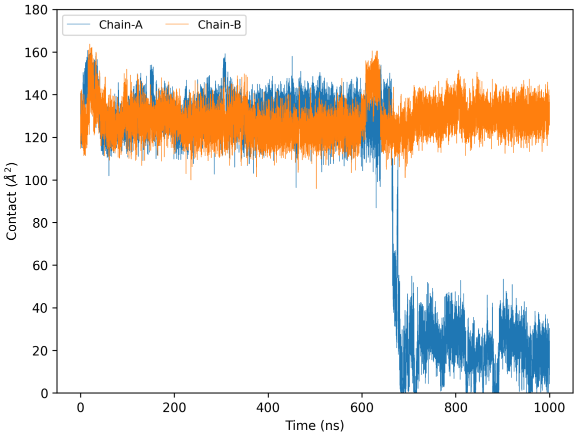

(d) Sim-03

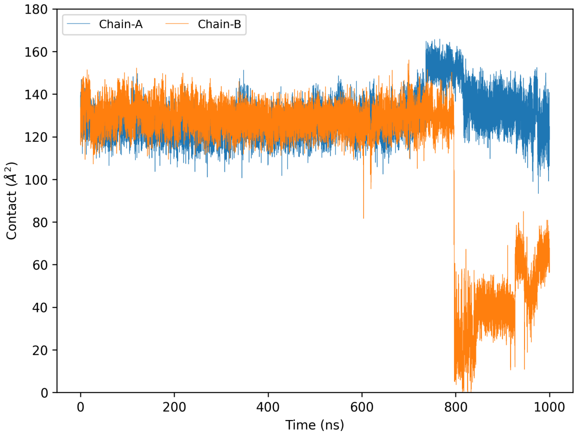

(e) Sim-04

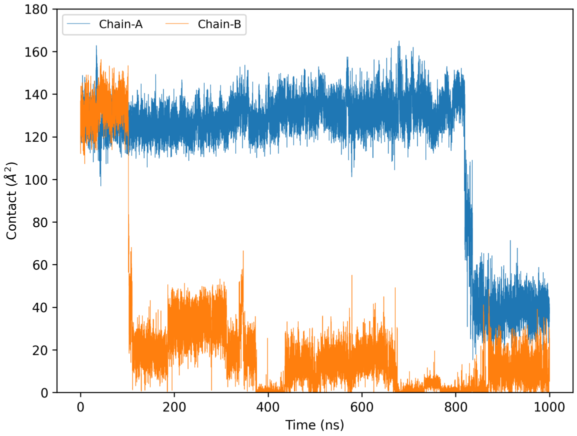

(f) Sim-05

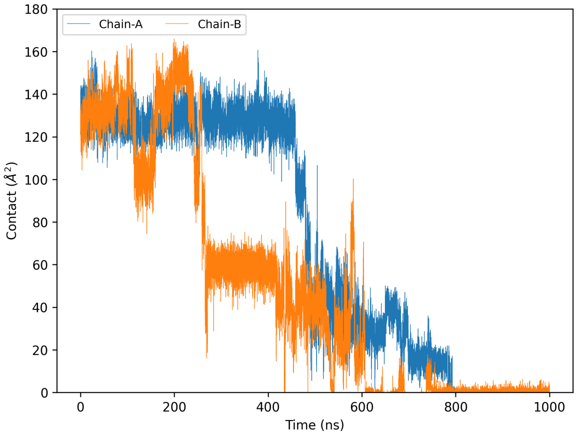

(g) Sim-06

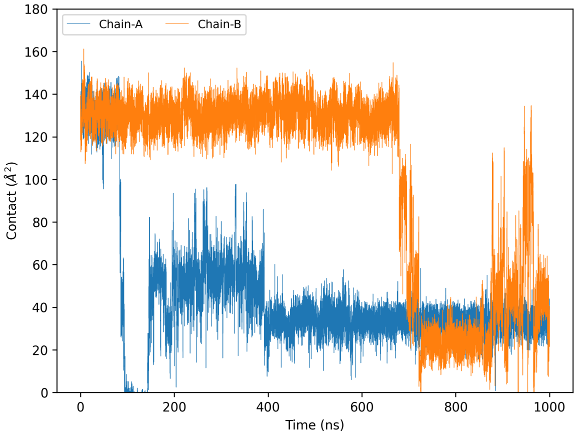

(h) Sim-07

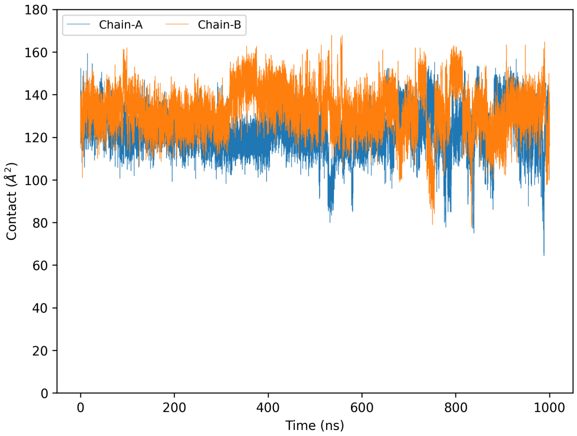

(i) Sim-08

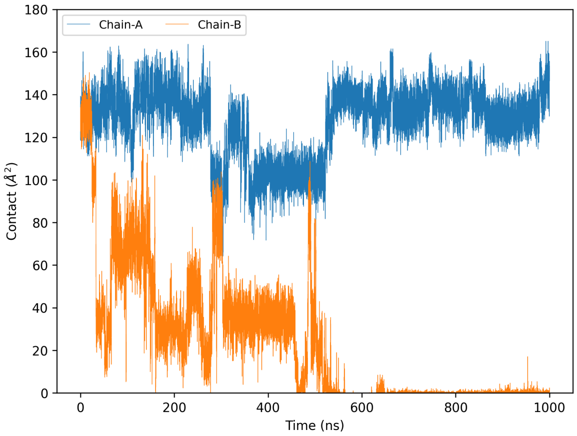

(j) Sim-09

Figure S9: Surface Contact analysis between ligand and active site residues during the 10 PS-V simulations.

Linear Interaction Energy (LIE) Analysis of PS-I and PS-V

Scripts for Data Preparation

```
#!/bin/bash

function trajin()
{
for j in {00..09}
    do
        cat > ${fileName}-${j}.in << END
parm ${dir}/${j}/md.top
trajin ${dir}/${j}/sampling-???.nc

lie LIE_His_Arg ":455 & ! @CA,HA,C,O,N,H" ":653 & ! @CA,HA,C,O,N,H" out ${fileName}-A-{j}.dat
lie LIE_His_Arg ":1109 & ! @CA,HA,C,O,N,H" ":1307 & ! @CA,HA,C,O,N,H" out ${fileName}-B-{j}.dat
run
END
        cpptraj.OMP -i ${fileName}.in
done
}

rootPath=/mnt/beegfs/ese86/shared/Erdem

fileName=ps1-lie dir=${rootPath}/CYS-HIE-ASP trajin
fileName=ps5-lie dir=${rootPath}/CYS-HIP-ASP trajin
```

LIE Plots of PS-I System

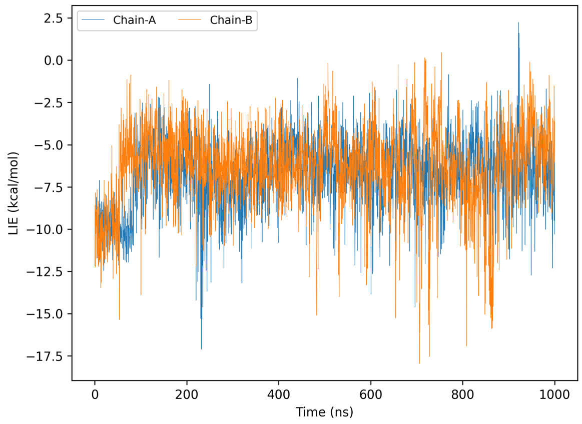

(a) Sim-00

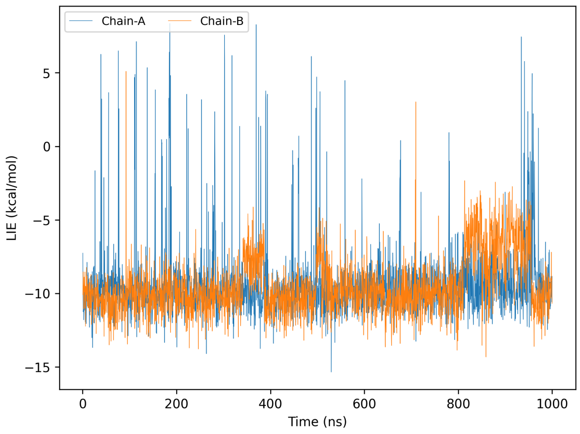

(b) Sim-01

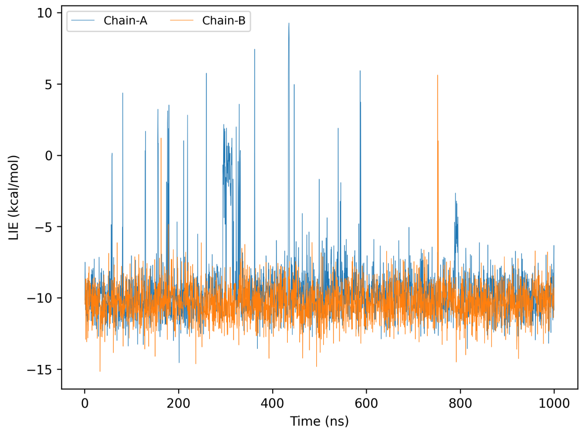

(c) Sim-02

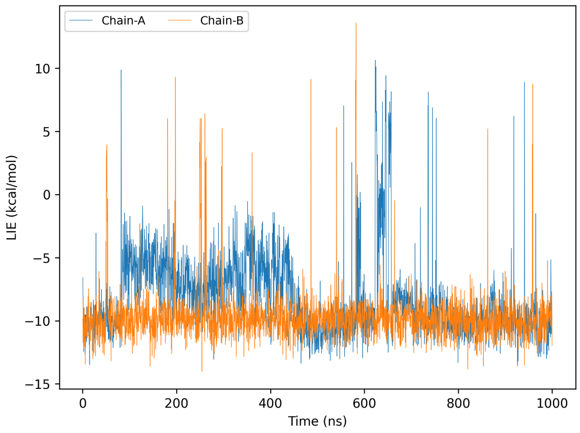

(d) Sim-03

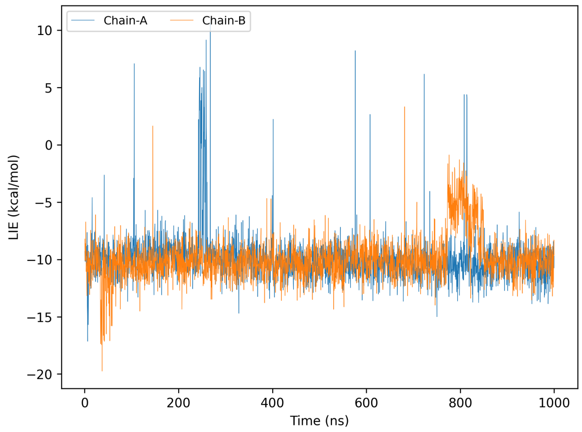

(e) Sim-04

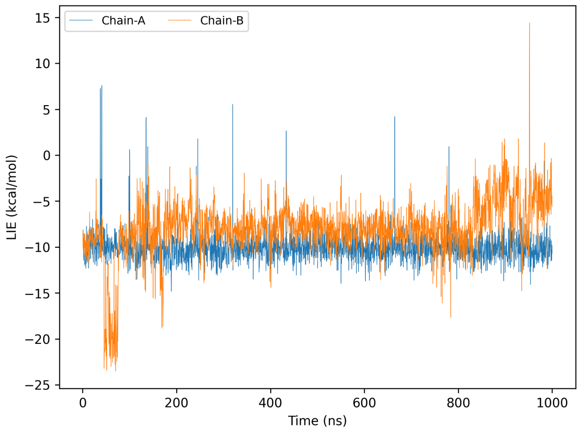

(f) Sim-05

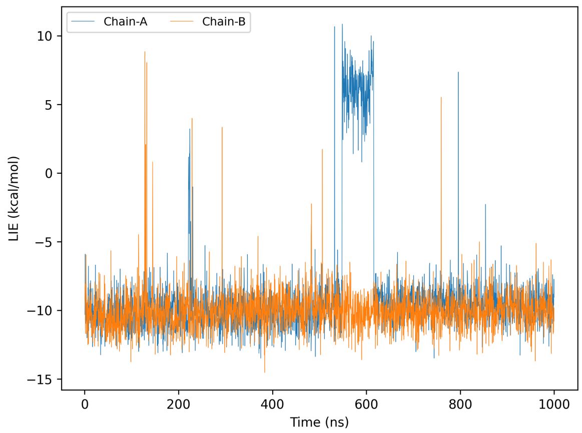

(g) Sim-06

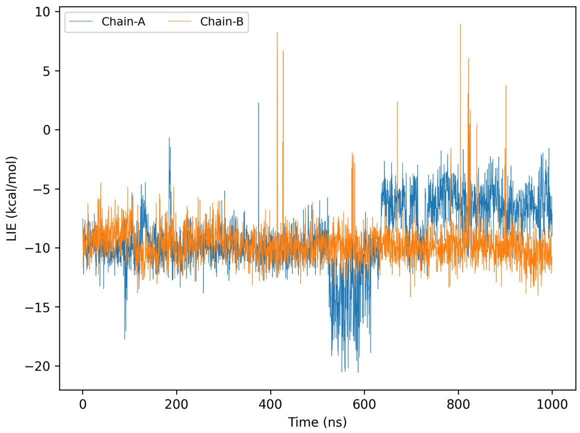

(h) Sim-07

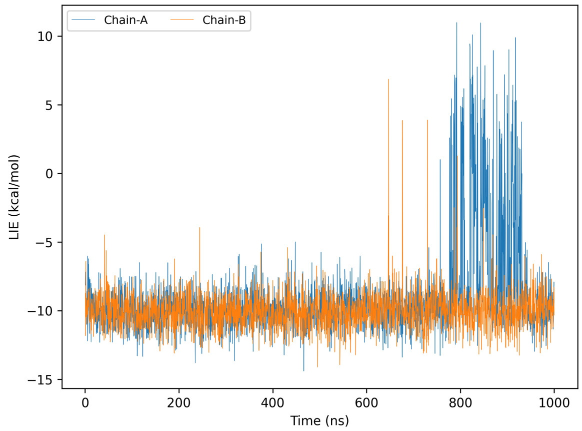

(i) Sim-08

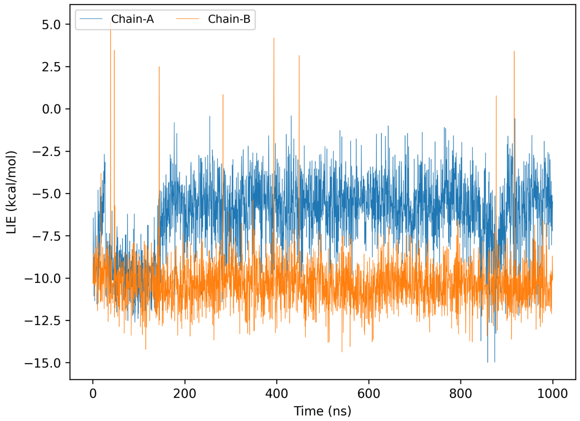

(j) Sim-09

Figure S10: LIE analysis between ligand Arg residue and HIS471 residue during the 10 PS-I simulations.

LIE Plots of PS-V System

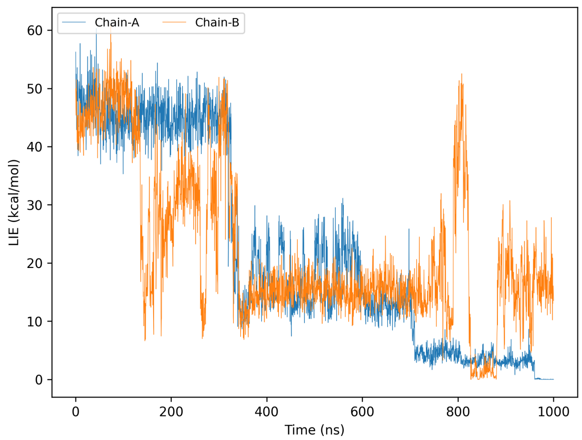

(a) Sim-00

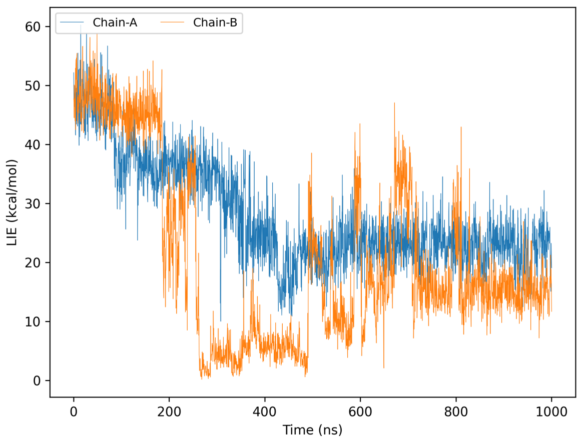

(b) Sim-01

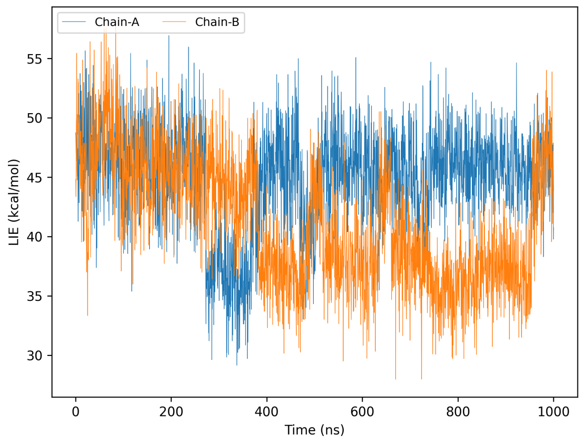

(c) Sim-02

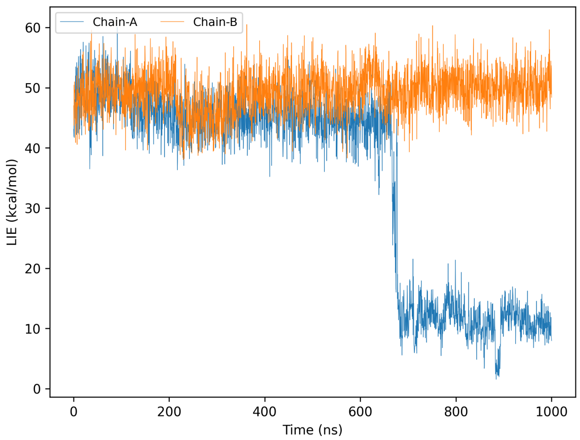

(d) Sim-03

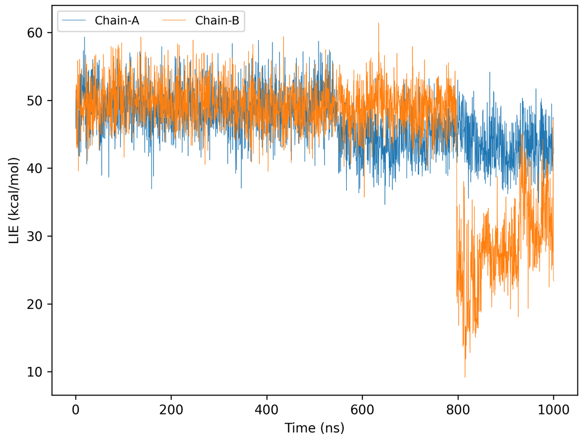

(e) Sim-04

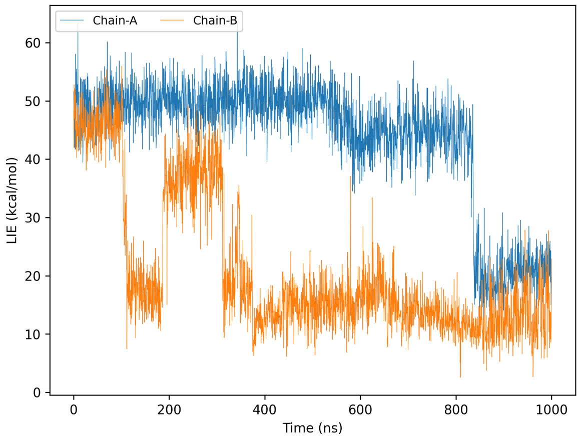

(f) Sim-05

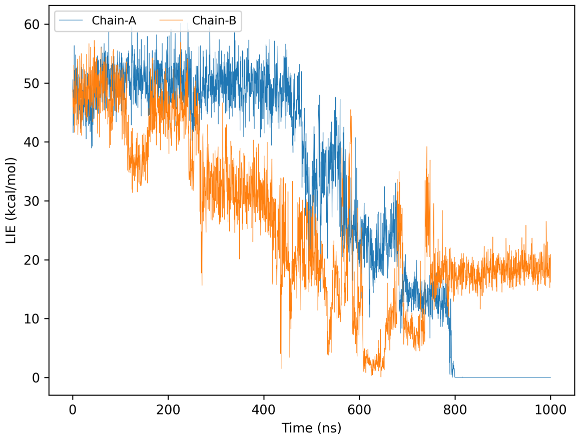

(g) Sim-06

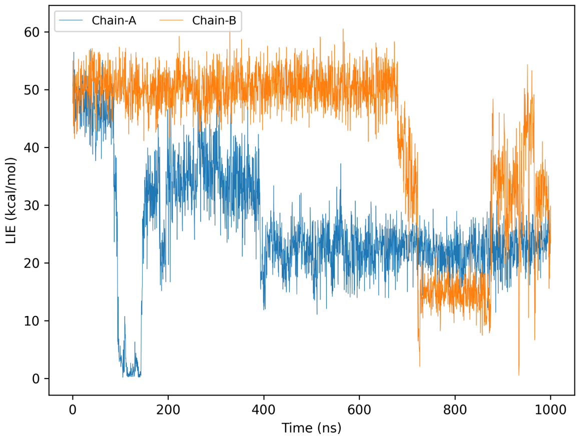

(h) Sim-07

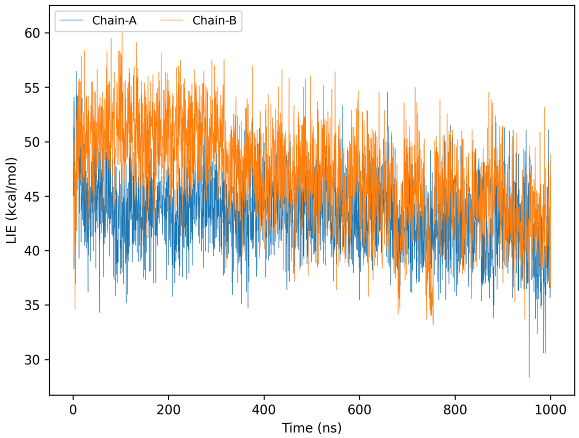

(i) Sim-08

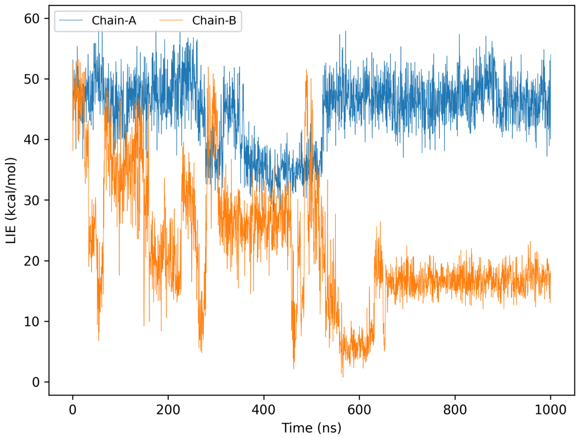

(j) Sim-09

Figure S11: LIE analysis between ligand Arg residue and HIS471 residue during the 10 PS-V simulations.

Near Attack Conformer (NAC) Analysis of PS-I

Scripts for Data Preparation

```bash
#!/bin/bash

declare -A res
res+=(  ["cysA"]=7 ["cysB"]=7
        ["argA"]=8 ["argB"]=8
        ["asp1A"]=5 ["asp1B"]=5
        ["asp2A"]=1 ["asp2B"]=1
        ["hisA"]=3 ["hisB"]=3 )

function trajin()
{
for j in {00..09};
do
   for k in A B;
   do
      cat > ${fileName}-${j}-${k}.in << END
parm ${dir}/${j}/md.top
trajin ${dir}/${j}/sampling-???.nc

##Stepwise
distance d-SG-CZ  :${res[cys${k}]}@SG :${res[arg${k}]}@CZ out ${fileName}-${j}-${k}.dat #2
distance d-HG-OD1 :${res[cys${k}]}@HG :${res[asp1${k}]}@OD1 out ${fileName}-${j}-${k}.dat #3
distance d-HG-OD2 :${res[cys${k}]}@HG :${res[asp1${k}]}@OD2 out ${fileName}-${j}-${k}.dat #4
angle a-SG-HG-OD1 :${res[cys${k}]}@SG :${res[cys${k}]}@HG :${res[asp1${k}]}@OD1 out ${fileName}-${j}-${k}.dat #5
angle a-SG-HG-OD2 :${res[cys${k}]}@SG :${res[cys${k}]}@HG :${res[asp1${k}]}@OD2 out ${fileName}-${j}-${k}.dat #6

##Stepwise water assisted
distance d-HG-WO   :${res[cys${k}]}@HG :WAT@O out ${fileName}-${j}-${k}.dat #7
distance d-WH1-OD1 :WAT@H1 :${res[asp1${k}]}@OD1 out ${fileName}-${j}-${k}.dat #8
distance d-WH1-OD2 :WAT@H1 :${res[asp1${k}]}@OD2 out ${fileName}-${j}-${k}.dat #9
distance d-WH2-OD1 :WAT@H2 :${res[asp1${k}]}@OD1 out ${fileName}-${j}-${k}.dat #10
distance d-WH2-OD2 :WAT@H2 :${res[asp1${k}]}@OD2 out ${fileName}-${j}-${k}.dat #11

angle a-SG-HG-WO   :${res[cys${k}]}@SG :${res[cys${k}]}@HG :WAT@O out ${fileName}-${j}-${k}.dat #12
angle a-WO-WH1-OD1 :WAT@O :WAT@H1 :${res[asp1${k}]}@OD1 out ${fileName}-${j}-${k}.dat #13
angle a-WO-WH1-OD2 :WAT@O :WAT@H1 :${res[asp1${k}]}@OD2 out ${fileName}-${j}-${k}.dat #14
```

```
angle a-WO-WH2-OD1 :WAT@O :WAT@H2 :${res[asp1${k}]}@OD1 out ${fileName}-${j}-${k}.dat #15
angle a-WO-WH2-OD2 :WAT@O :WAT@H2 :${res[asp1${k}]}@OD2 out ${fileName}-${j}-${k}.dat #16

#Concerted
distance d-HG-NH1  :${res[cys${k}]}@HG :${res[arg${k}]}@NH1 out ${fileName}-${j}-${k}.dat #17
distance d-HG-NH2  :${res[cys${k}]}@HG :${res[arg${k}]}@NH2 out ${fileName}-${j}-${k}.dat #18
dihedral di-SG-HG-NH1-CZ :${res[cys${k}]}@SG :${res[cys${k}]}@HG :${res[arg${k}]}@NH1 \
                         :${res[arg${k}]}@CZ out ${fileName}-${j}-${k}.dat #19
dihedral di-SG-HG-NH2-CZ :${res[cys${k}]}@SG :${res[cys${k}]}@HG :${res[arg${k}]}@NH2 \
                         :${res[arg${k}]}@CZ out ${fileName}-${j}-${k}.dat #20

##Concerted water assisted
distance d-WH1-NH1 :WAT@H1 :${res[arg${k}]}@NH1 out ${fileName}-${j}-${k}.dat #21
distance d-WH1-NH2 :WAT@H1 :${res[arg${k}]}@NH2 out ${fileName}-${j}-${k}.dat #22
distance d-WH2-NH1 :WAT@H2 :${res[arg${k}]}@NH1 out ${fileName}-${j}-${k}.dat #23
distance d-WH2-NH2 :WAT@H2 :${res[arg${k}]}@NH2 out ${fileName}-${j}-${k}.dat #24

angle a-WO-WH1-NH1 :WAT@O :WAT@H1 :${res[arg${k}]}@NH1 out ${fileName}-${j}-${k}.dat #25
angle a-WO-WH1-NH2 :WAT@O :WAT@H1 :${res[arg${k}]}@NH2 out ${fileName}-${j}-${k}.dat #26
angle a-WO-WH2-NH1 :WAT@O :WAT@H2 :${res[arg${k}]}@NH1 out ${fileName}-${j}-${k}.dat #27
angle a-WO-WH2-NH2 :WAT@O :WAT@H2 :${res[arg${k}]}@NH2 out ${fileName}-${j}-${k}.dat #28

run

END
cpptraj.OMP -i ${fileName}-${j}-${k}.in
   done
done
}

rootPath=/mnt/beegfs/ese86/shared/Erdem

fileName=ps1-nac dir=${rootPath}/CYS-HIE-ASP trajin
```

NAC Plots of PS-I System

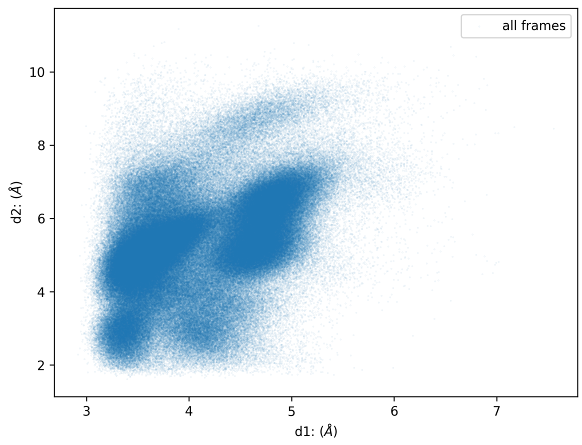

(a) Stepwise: All Frames

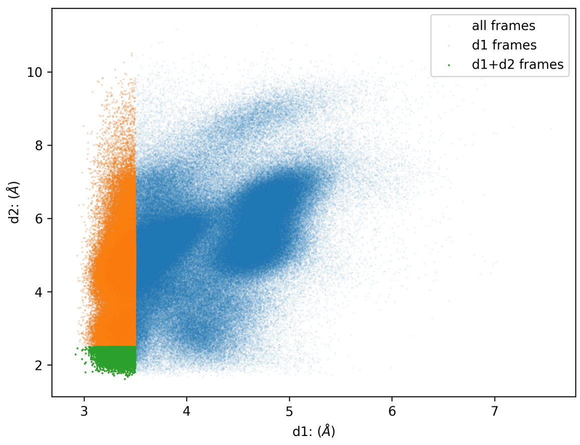

(b) Stepwise: NAC Frames

Figure S12: NAC frames of reaction mechanism 1 (Stepwise). Blue markers present all frames (500000 frames), Orange markers present frames filtered by d1 distance criteria (91180 frames) and green markers present frames filtered by d1 and d2 distance criterias (4651 frames)

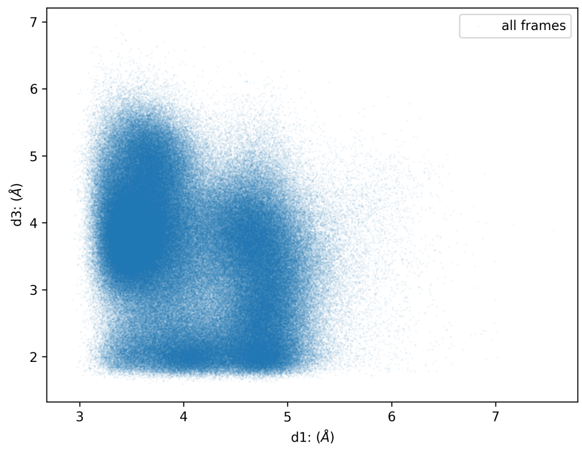

(a) Stepwise water assisted: All Frames

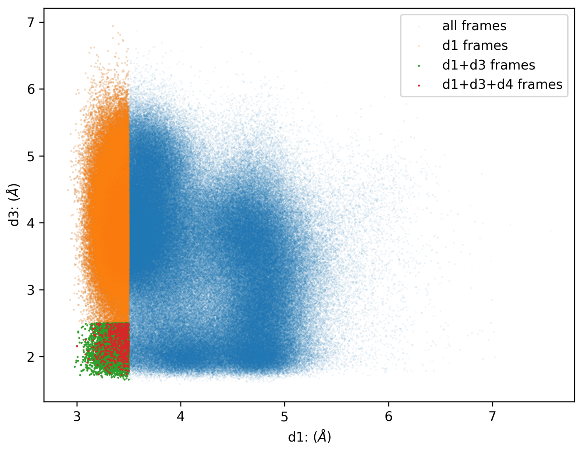

(b) Stepwise water assisted: NAC Frames

Figure S13: NAC frames of reaction mechanism 2 (Stepwise water assisted). Blue markers present all frames (500000 frames), Orange markers present frames filtered by d1 distance criteria (91180 frames), green markers present frames filtered by d1 and d3 distance criterias (6472 frames), and red markers present frames filtered by d1, d3 and d4 distance criterias (684 frames)

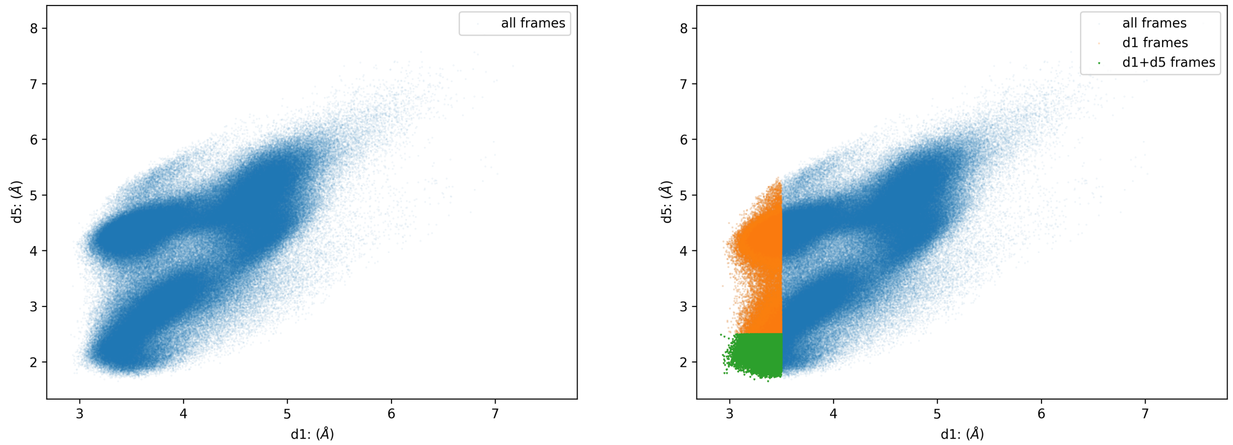

(a) Concerted: All Frames (b) Concerted: NAC Frames

Figure S14: NAC frames of reaction mechanism 3 (Concerted). Blue markers present all frames (500000 frames), Orange markers present frames filtered by d1 distance criteria (91180 frames) and green markers present frames filtered by d1 and d5 distance criterias (27042 frames)

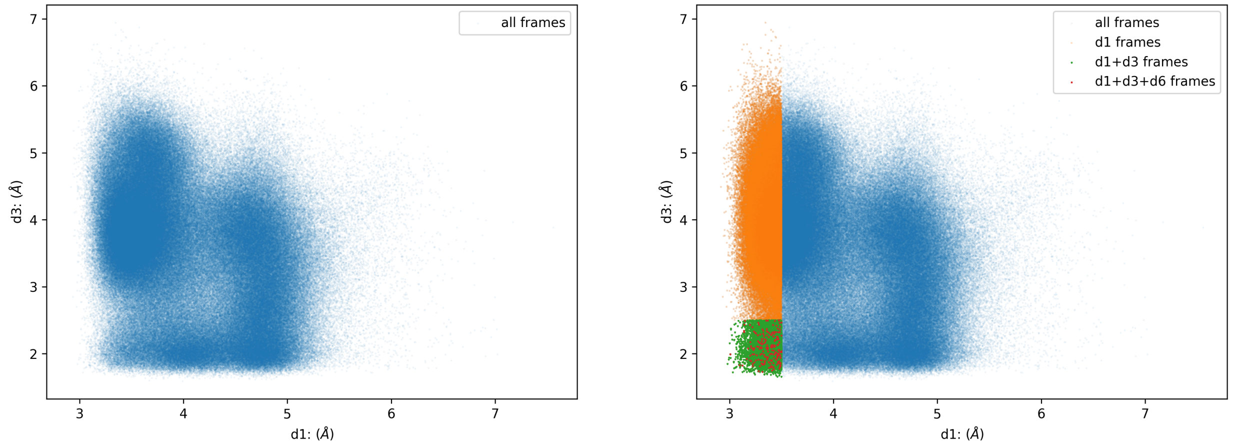

(a) Concerted water assisted: All Frames (b) Concerted water assisted: NAC Frames

Figure S15: NAC frames of reaction mechanism 4 (Concerted water assisted). Blue markers present all frames (500000 frames), Orange markers present frames filtered by d1 distance criteria (91180 frames), green markers present frames filtered by d1 and d3 distance criterias (6472 frames), and red markers present frames filtered by d1, d3 and d6 distance criterias (188 frames)

Interaction between Substrate and ASP351

Scripts for Data Preparation

```
#!/bin/bash

declare -A res
res+=(["cysA"]=627 ["cysB"]=1281
["argA"]=653 ["argB"]=1307
["asp1A"]=457 ["asp1B"]=1111
["asp2A"]=335 ["asp2B"]=989
["hisA"]=455 ["hisB"]=1109)

function trajin()
{
for j in {00..09};
do
   cat > ${fileName}-${j}-${k}.in << END
parm ${dir}/${j}/md.top
trajin ${dir}/${j}/sampling-???.nc
END

   for o in OD1 OD2;
   do
      for h in HE HH21 HH22;
      do
         cat >> ${fileName}-${j}-${k}.in << END
distance d-${o}-${h}-A    :${res[asp2A]}@${o} :${res[argA]}@${h} out ${fileName}-${j}-${k}.dat
distance d-${o}-${h}-B    :${res[asp2B]}@${o} :${res[argB]}@${h} out ${fileName}-${j}-${k}.dat
END
      done
   done
run

cpptraj.OMP -i ${fileName}-${j}-${k}.in
done

}

rootPath=/mnt/beegfs/ese86/shared/Erdem
```

```
fileName=ps1-asp351 dir=${rootPath}/CYS-HIE-ASP trajin
```

Histogram Plots of Minimum Distance Between ASP351@OD* atoms and Substrate H-atoms

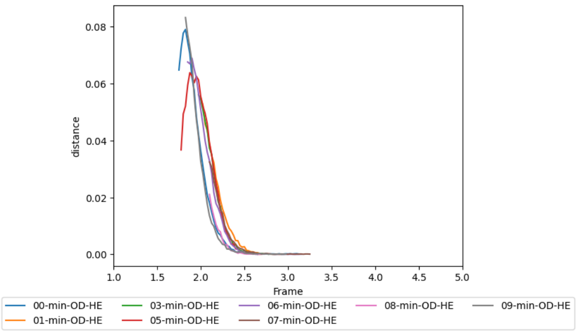

(a) Min Distance ASP351@OD-Substrate@HE for chain A

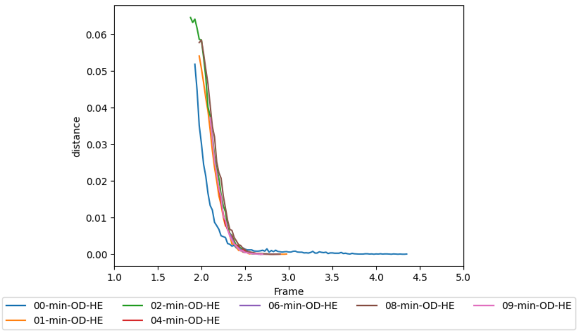

(b) Min Distance ASP351@OD-Substrate@HE for chain B

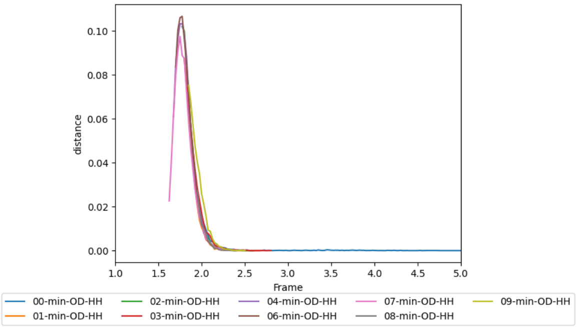

(c) Min Distance ASP351@OD-Substrate@HH for chain A

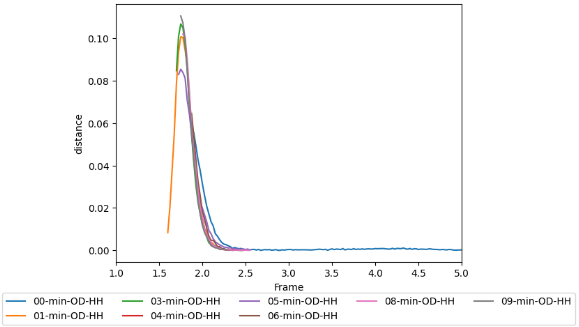

(d) Min Distance ASP351@OD-Substrate@HH for chain B

Figure S16: Distance analysis between ligand Arg residue and ASP351 residue during the 10 PS-I simulations.

Interaction between Substrate and ASP473

Scripts for Data Preparation

```
#!/bin/bash

declare -A res
res+=(["cysA"]=627 ["cysB"]=1281
["argA"]=653 ["argB"]=1307
["asp1A"]=457 ["asp1B"]=1111
["asp2A"]=335 ["asp2B"]=989
["hisA"]=455 ["hisB"]=1109)

function trajin()
{
for j in {00..09};
do
   for k in A B;
   do
      cat > ${fileName}-${j}-${k}.in << END
parm ${dir}/${j}/md.top
trajin ${dir}/${j}/sampling-???.nc
END

      for o in OD1 OD2;
      do
         for h in HH12 HH22;
         do
            cat >> ${fileName}-${j}-${k}.in << END
distance d-${o}-${h}    :${res[asp1${k}]}@${o} :${res[arg${k}]}@${h} out ${fileName}-${j}-${k}.dat
END
         done
     done

     echo "hbond donormask :${res[arg${k}]}@HH12,HH22 acceptormask :${res[asp1${k}]}@OD1,OD2 out ${fileName}-${j}-${k}-nhb.dat >
      avgout > ${fileName}-${j}-${k}-avghb.dat" >> ${fileName}-${j}-${k}.in

     echo "run" >> ${fileName}-${j}-${k}.in

     cpptraj.OMP -i ${fileName}-${j}-${k}.in
```

```
   done
done
}


function trajin_rdf()
{
cpptraj.OMP << END
parm ${dir}/00/md.top
trajin ${dir}/${j}/sampling-???.nc
autoimage :627
radial out radial.dat 0.03 12.0  :653,1307@HH=  :457,1111@OD1,OD2 intrdf radial.dat
END
}

rootPath=/mnt/beegfs/ese86/shared/Erdem

fileName=ps1-asp473-sub dir=${rootPath}/CYS-HIE-ASP trajin

dir=${rootPath}/CYS-HIE-ASP trajin_rdf
```

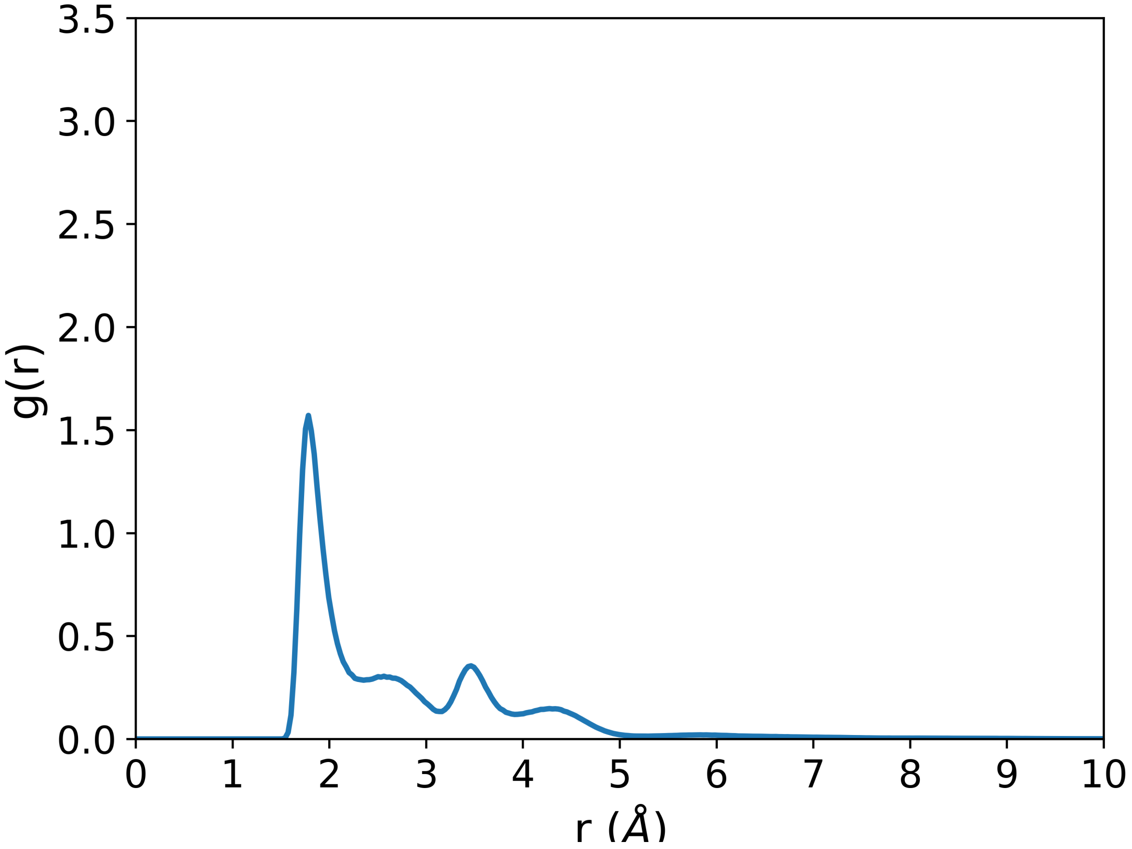

Figure S17: interaction between ARG623@HH12,HH22-ASP473@OD1,OD2 for the system PS-I
